# Cell-free microbial culture filtrates as candidate biostimulants to enhance plant growth and yield and activate soil- and plant-associated beneficial microbiota

**DOI:** 10.3389/fpls.2022.1040515

**Published:** 2022-12-23

**Authors:** Rafael Jorge León Morcillo, Edurne Baroja-Fernández, Lidia López-Serrano, Jesús Leal-López, Francisco José Muñoz, Abdellatif Bahaji, Alberto Férez-Gómez, Javier Pozueta-Romero

**Affiliations:** ^1^ Institute for Mediterranean and Subtropical Horticulture “La Mayora” (IHSM), Consejo Superior de Investigaciones Científicas-Universidad de Málaga, Málaga, Spain; ^2^ Instituto de Agrobiotecnología (IdAB), Consejo Superior de Investigaciones Científicas-Gobierno de Navarra, Nafarroa, Spain

**Keywords:** plant-microbe interaction, biostimulants, crop yield, stress tolerance, volatile organic compounds, soil- and plant-associated microbiota

## Abstract

In this work we compiled information on current and emerging microbial-based fertilization practices, especially the use of cell-free microbial culture filtrates (CFs), to promote plant growth, yield and stress tolerance, and their effects on plant-associated beneficial microbiota. In addition, we identified limitations to bring microbial CFs to the market as biostimulants. In nature, plants act as metaorganisms, hosting microorganisms that communicate with the plants by exchanging semiochemicals through the phytosphere. Such symbiotic interactions are of high importance not only for plant yield and quality, but also for functioning of the soil microbiota. One environmentally sustainable practice to increasing crop productivity and/or protecting plants from (a)biotic stresses while reducing the excessive and inappropriate application of agrochemicals is based on the use of inoculants of beneficial microorganisms. However, this technology has a number of limitations, including inconsistencies in the field, specific growth requirements and host compatibility. Beneficial microorganisms release diffusible substances that promote plant growth and enhance yield and stress tolerance. Recently, evidence has been provided that this capacity also extends to phytopathogens. Consistently, soil application of microbial cell-free culture filtrates (CFs) has been found to promote growth and enhance the yield of horticultural crops. Recent studies have shown that the response of plants to soil application of microbial CFs is associated with strong proliferation of the resident beneficial soil microbiota. Therefore, the use of microbial CFs to enhance both crop yield and stress tolerance, and to activate beneficial soil microbiota could be a safe, efficient and environmentally friendly approach to minimize shortfalls related to the technology of microbial inoculation. In this review, we compile information on microbial CFs and the main constituents (especially volatile compounds) that promote plant growth, yield and stress tolerance, and their effects on plant-associated beneficial microbiota. In addition, we identify challenges and limitations for their use as biostimulants to bring them to the market and we propose remedial actions and give suggestions for future work.

## Introduction

Plant’ growth and development are influenced by microorganisms occurring in the phytosphere that communicate with plants by exchanging chemical signals (Hartmann et al., 2014). Some of these microorganisms can benefit host plants in a variety of ways, a scenario of utmost interest when searching for new and efficient agricultural approaches based on manipulation of plant-associated microbiota. Beneficial microorganisms can directly promote plant growth through mechanisms involving production of bioactive compounds (e.g. phytohormones, volatile compounds, peptides, etc.), dinitrogen fixation, solubilization of minerals and organic material and enhancement of water and nutrient uptake and use (Tsavkelova et al., 2006a; Rodríguez et al., 2007; Francis et al., 2010). These microorganisms can also indirectly promote plant growth by antagonism/antibiosis against pathogens, alleviation of stress caused by environmental pollutants or other stressful abiotic conditions (e.g. drought and salinity), or by triggering in the host plant enhanced defense capacities against pathogen attack.

A decline in natural resources and the environmental damage caused by practices relying on the excessive and inappropriate application of fertilizers and depletion of soil and water resources have become major limitations in conventional agriculture. A more sustainable and eco-friendly agriculture requires increases in product yield quality, while reducing the negative environmental impact of agrochemicals on soil fertility and biodiversity; potential solutions may be fostered by microbial-based approaches (Calvo et al., 2014). The aim of this review was to compile information on current and emerging microbial-based fertilization practices, particularly the use of microbial inoculants, microbial-derived compounds and microbial culture filtrates (CFs), to promote plant growth, yield and stress tolerance, and their effects on plant-associated beneficial microbiota. In addition, we identify challenges and limitations to bring microbial CFs to the market as biostimulants compliant with scientific requirements of the official regulations for fertilizer products.

## Soil inoculation of beneficial microorganisms: limitations of a widely used practice to enhance crop yield and/or protect plants from (a)biotic stresses in an eco-friendly manner

One environmentally safe and sustainable practice to promote plant growth, increase crop yield and/or enhance stress tolerance is based on the inoculation of soil with plant growth promoting microorganisms (PGPM) (Miransari, 2011; Calvo et al., 2014; Ahmad et al., 2018; Backer et al., 2018; Fiorentino et al., 2018; Zhong et al., 2019; Noceto et al., 2021). Microbial inoculants consist of one or a reduced number of microbial strains which are grown separately or in mixed culture fermentation, concentrated and then formulated with an appropriate carrier into the final product form. Despite its undisputable success (Li et al., 2022), the technology based on PGPM inoculation has faced a number of limitations and inconsistences that are summarized in **Table 1**. First, the efficiency of inoculation depends on soil pH, temperature and nutrient content, interaction with the crop species (host specificity), competition with native strains and compatibility between the microbial inoculants (Svenningsen et al., 2018; Emmanuel and Babalola, 2020). Second, development of multi-strain bioinoculants on a large-scale level is difficult since each co-inoculant requires specific culture media and physical-chemical conditions (Reddy and Saravanan, 2013). Third, inoculation with beneficial microorganisms without a proper carrier or formulation may result in a rapid decline in the applied microbial population, as the inoculated microbes must compete with the often better-adapted native microbial community (Bashan et al., 2014; Cardinale et al., 2015; Parnell et al., 2016). For instance, the abundance in soil of some well-known beneficial microbes such as *Bacillus amyloliquefaciens* FZB42 and *Trichoderma harzianum* falls below detection limits just a few weeks after application (Papavizas, 1982; Kröber et al., 2014; Oskiera et al., 2017). Although seed coating with beneficial microorganisms may be a suitable option to maintain microbial survival in soil over a longer period, an appropriate coating requires a delicate balance between coating materials, microbe and compatible chemistry, which is not always easy to obtain (Glare et al., 2012; Parnell et al., 2016). Moreover, an adequate delivery system for microorganisms in the soil is also required, which represents a major challenge to industry since it involves mass production, formulation, and application of the beneficial microbes (Ravensberg, 2011; Glare et al., 2012; Vassilev et al., 2020). Fourth, some PGPM including some *Pseudomonas* spp. are opportunistic pathogens (Belimov et al., 2007; Sitaraman, 2015) and thus their use is associated with a pathogenicity risk. Fifth, the growth-promoting effect of inoculating beneficial microorganisms strongly depends on the nutritional status of the plant in relation to the extent that they can be rendered ineffective in promoting plant growth (Hoeksema et al., 2010; Pineda et al., 2013). Thus, plants respond better to mycorrhizal inoculation when grown in soils with high microbial diversity and when subjected to phosphorous limitation (Hoeksema et al., 2010). For instance, *Colletotrichum tofieldiae* promotes growth in *Arabidopsis thaliana* only under phosphorus deficiency conditions (Hiruma et al., 2016). Inoculation with the growth-promoting rhizobacterium *B. amyloliquefaciens* GB03 can have deleterious effects on plant growth under phosphate deficiency conditions, due to an activation of the phytohormone-mediated immune response modulated by a phosphate-starvation response (Morcillo et al., 2020). Sixth, the PGPM inoculation efficiency largely depends upon production of bioactive compounds by the inoculated microbes, which in turn strongly depends on abiotic and biotic environmental contexts. Therefore, it can never be guaranteed that inoculation of a particular microbe will result in the production of compounds with plant growth-promoting or stress tolerance-conferring properties. Seventh, most of soil and plant-associated microorganisms cannot be cultured in reactors. Eighth, inoculation of non-native, allochthonous microorganisms is known to produce strong shifts in microbial communities (Schmidt et al., 2014; Diagne et al., 2018; Berg et al., 2021), with unpredictable and unwanted effects (Hart et al., 2018). For instance, non-native mycorrhizal fungal commercial inoculants may lead to undesirable promotion of exotic over native plant species (Burkle and Belote, 2015; Middleton et al., 2015; Hart et al., 2018).

**Table 1:** Limitations of soil inoculation of PGPM and application of microbial-derived compounds and microbial culture filtrates.

	PGPM inoculation	Application of microbial-derived compounds	Application of microbial culture filtrates
Efficiency depends on environmental factors, host compatibility and competition with native microbes	x		
Efficiency depends on microbial culture composition and age	x	x	x
Difficulty to develop multi-strain bioinoculants	x		
Manner of application: necessity for a proper carrier or formulation	x	x	x
Pathogenicity risk to indigenous microbial communities and/or plants	x		
Biostimulant effect depends on plant nutritional status	x		
Scaling-up: difficulty to culture plant-associated microbes in reactors	x	x	x
Long and difficult process to isolate, identify and purify the beneficial compound		x	
Dose-dependence response		x	x
Complex synergic and cooperative interactions between different compounds to promote plant growth		x	
Contradictory effect on different plant species		x	
Antagonistic effects on native microbiota		x	
High production cost		x	x

“X” highlights the limitation of each microbial-derived method.

Some of the constraints of the classic single and multi-strain bioinoculation approach can be circumvented by holistic approaches based on the use of SynComs (for **Syn**thetic microbial **Com**munitie**s**), which has emerged as a new paradigm not only to better understand plant-microbe interactions, but also to benefit from them (Castrillo et al., 2017; Durán et al., 2018; Kwak et al., 2018; Carrión et al., 2019; Marin et al., 2021). SynComs are based on the use of metagenomic tools to determine the structure and potential function of plant-associated microbial communities, followed by the isolation and co-culturing of multiple locally adapted native microorganisms. Establishment and survival of inoculated SynComs in the field are higher than that of single or multi-strain bioinoculations, as SynComs are capable of competing with the pre-existing microbiota present in the plant or soil (Liu et al., 2022; Shayanthan et al., 2022). However, despite the obvious theoretical advantages of application of SynComs designed “à la carte” to mimic the role of a particular microbiome, this technology still has some constraints, including technical limitations in the correct metagenomic identification of the isolated microorganisms (Liu et al., 2020), development of SynComs inoculants at a large-scale industrial level, dependence upon the nutritional status of the plant, maintenance of the stability and function of SynComs over time under changing environmental conditions in open field, etc.

## Application of microbial-derived compounds: A step to minimize shortfalls related to PGPM inoculation technology

Depending on environmental conditions, microorganisms can release diffusible compounds including phytohormones, siderophores, proteins, peptides, sugar-derived molecules, amino acids, exopolysaccharides, organic acids and volatile compounds that alter metabolism, enhance photosynthesis, promote plant growth, confer resistance to (a)biotic stresses and cause massive lateral root formation, thus improving the root´s exploratory capacity for nutrients and predisposing plants for colonization and infection by microbes (Ryu et al., 2003; Arkhipova et al., 2005; Tsavkelova et al., 2006b; Spaepen et al., 2007; Berg, 2009; Contreras-Cornejo et al., 2009; Ortíz-Castro et al., 2009; Chanclud and Morel, 2016; Saha et al, 2016; Sánchez-López et al, 2016; Egamberdieva et al., 2017; García-Gómez et al., 2019; Morcillo and Manzanera, 2021). Some of these compounds are capable of activating soil microbial activity (Rodríguez-Morgado et al., 2017; Macias-Benitez et al, 2020). To address limitations related to PGPM inoculation technology, the application of small quantities of microbial bioactive compounds in pure form, either as alternatives, supplements or complements to microbial cells, has been proposed as a possible approach for improving crop productivity and stress tolerance while reducing agrochemical use (Kanchiswamy et al., 2015; Naamala and Smith, 2021). This approach offers reliability and the easy control of the quantity and quality of a compound of interest (**Table 1**). Furthermore, compared with the PGPM inoculation technology, the use of pure microbial compounds can benefit a broader range of crops and minimize pathogenicity risk (**Table 1**). Although the stability of many of these compounds depends on abiotic environmental factors (temperature, salt concentration in soil, pH, etc.) and biotic factors (they could be used by native microorganisms as nutrient source), their rapid perception by plants can prime them for growth promotion. However, there are quite a number of limitations associated with the use of microbe-derived compounds including time-consuming processes of isolation, identification and purification of bioactive compounds, dose-dependence of the response, complex synergic and cooperative interactions between different compounds to promote plant growth, contradictory effects of the same compound on different plants, antagonistic effects on beneficial microbiota, etc. (Naamala and Smith, 2021) (**Table 1**).

## Application of cell-free microbial culture filtrates: A sustainable and environmentally friendly approach to activate the soil- and plant-associated beneficial microbiota and cope with constraints related to PGPM inoculation and application of microbial-derived compounds?

Some of the limitations related to the use of microbe-derived compounds could be circumvented by the use of cell-free filtrates of beneficial bacterial and fungal cultures, which are mixtures of phytohormones, siderophores, proteins, peptides, amino acids, exopolysaccharides, organic acids, volatile compounds, etc. derived from broth cultures processed through centrifugation or filtration (i.e. micro/ultra/nanofiltration and inverse osmosis) for cell removal (Pellegrini et al., 2020). Agronomic studies have provided evidence that application of these complex cocktails is an efficient approach to promote plant growth and enhance yield and stress tolerance in a wide range of crops while reducing the use of agrochemicals. Notably, recent studies have shown that cell-free CFs of phytopathogens can also be used to enhance yield and stress tolerance (Baroja-Fernández et al., 2021 and unpublished results). **Table 2** summarizes details of the studies on effects of microbial CF application. Most of these studies indicated that phytohormones occurring in the microbial CFs (especially indole acetic acid (IAA)) are major determinants of the response of plants to these extracts. However, some studies indicated that microbial amino acids, peptides, extracellular proteins, lipopeptides and siderophores could also play important roles in the response of plants to fungal CFs. For instance, Buensateai et al. (2013) showed that application of *Bacillus* sp. strain CaSUT007 CFs enriched in extracellular proteins increased root and shoot lengths and total biomass of cassava stalks. Furthermore, Posada et al. (2016) showed that application of *B. subtilis* EA-CB0575 CFs enhanced dry weight of banana plants by the action of lipopeptides and siderophores. Moreover, Buensanteai et al. (2008) showed that extracts of *B. amyloliquefaciens* strain KPS46 promoted soybean growth through the actions of the antibiotic surfactin and proteins secreted by the bacterium, including auxin biosynthetic enzymes, proteins related to phosphate solubilization and nitrogen metabolism, antifungal lipopeptides and proteins related to protection against oxidative stress. Also, CFs enriched in amino acids secreted by several *Penicillium* spp. enhanced shoot and root length as well as the biomass of sesame plants under well-irrigated and drought conditions (Radhakrishnan et al., 2014).

**Table 2:** Studies on effects of microbial CF application on plant.

Microbial species		Culture medium	Plant species	Application manner	Effect on plant	Mechanism/mode of action	Reference
*Bacterial species*							
***Azotobacter vinelandii***	Beneficial	Specific medium	*Solanum lycopersicum*	Root irrigation with culture supernatants	Increase shoot dry weight and fruit production	CFs contain auxins, gibberellins and cytokinin-like substances	Azcón and Barea (1975)
***Azotobacter beijerinckii***							
***Streptomyces olivaceoviridis***	Beneficial	Starch-casein medium	*Triticum aestivum*	Pretreatment of wheat grain with CFs	Enhance growth vigor and crop yield	CFs contain auxins, gibberellins and cytokinin-like substances	Aldesuquy et al. (1998)
***Streptomyces rimosus***							
***Streptomyces rochei***							
***Streptomyces atroolivaceus***	Beneficial	MBGM	*Triticum aestivum*	Pretreatment of wheat grain with CFs	Increases the shoot length, fresh and dry mass, root fresh and dry mass but suppresses the depth of the root system	Effects probably caused by activity of plant growth regulators	El-Shanshoury (1989)
***Azospirillum brasilense***	Beneficial	NFb	*Oryza sativa*	CFs applied in hydroponic medium	Enhance root growth and development	CFs contain IAA	El-Khawas and Adachi (1999)
***Klebsiella pneumoniae***		NFDM					
***Bacillus amyloliquefaciens*** ***(FZB24, FZB42, FZB45)***	Beneficial	GNB	*Zea mays*	Coleoptiles incubated with CFs	Enhance length growth	CFs contain IAA	Idris et al. (2004)
***Bacillus subtilis FZB37***							
***Bacillus amyloliquefaciens* KPS46**	Beneficial	GNB	*Glycine max*	Pretreatment of seeds with CFs	Increases root and shoot length and plant biomass	IAA and extracellular proteins	Buensanteai et al (2008)
***Streptomyces coelicolor***	Beneficial	GYMA broth	*Triticum aestivum*	Seed coating with CFs	Improve plant growth under water-stress conditions	CFs contain IAA	Yandigeri et al. (2012)
***Streptomyces olivaceus***							
***Streptomyces geysiriensis***							
***Methylobacterium* spp.**	Beneficial	AMS	*Triticum aestivum*	Pretreatment of seeds with CFs	Enhances seed germination and seedling growth	CFs contain CKs	Meena et al. (2012)
***Bacillus* sp. CaSUT007**	Beneficial	GNB	*Manihot esculenta*	Preteatment of stakes with CFs	Increases root and shoot lengths	IAA and extracellular proteins	Bulgarelli et al. (2013)
***Bacillus subtilis* EA-CB0575**	Beneficial	TSB, SBM	*Musa* spp.	Pretreatment of germinated seeds with CFs	Increases shoot length and dry weight	CFs contain Lipopeptides and siderophores	Posada et al. (2016)
***Burkholderia seminalis***	Beneficial	Specific medium	*Solanum lycopersicum*	*In vitro* application of CFs	Increases seed germination	CFs contain IAA	Tallapragada et al. (2015)
***Enterococcus faecium***	Neutral	LB	*Cucumis melo*	Soil irrigation with CFs	Increases shoot and root lengths, plant fresh weight, and chlorophyll content	CFs contain IAA and GAs	Lee et al. (2015)
***Streptomyces* sp.**	Beneficial	TYB	*Solanum lycopersicum*	Soil irrigation with CFs	Enhances plant growth	Effects probably caused by IAA production	Kaur et al. (2019)
***Azospirillum brasilense*** **(*Ab–V5, Ab–V6*)**	Beneficial	DYGS	*Glycine max*	Spraying of leaves or seeds	Increase root nodulation and root development	Indolic compounds	Rondina et al. (2020)
***Pectobacterium carotovorum***	Pathogenic	LB	*Arabidopsis thaliana*	Soil irrigation with CFs	Increase shoot and root biomass but not production	Unkwnon	Ávila and Poveda (2022)
***Pseudomonas syringa***							
*Fungal species*							
***Piriformospora indica***	Beneficial	Specific medium	*Zea mays*	Root irrigation with CFs	Enhances shoot but not root growth	Unknown	Varma et al. (1999)
***Piriformospora indica***	Beneficial	M+ medium	*Arabidopsis thaliana*	*In vitro* application of CFs	Induces root branching	Production of a diffusible factor that is probably IAA	Sirrenberg et al. (2007)
***Sebacina vermifera***	Beneficial	MYP	*Panicum virgatum*	Pretreatment of seeds with CFs	Enhances seed germination and biomass production	Unknown	Ghimire et al. (2009)
***Cladosporium* sp. MH-6**	Beneficial	Czapek’s broth	*Cucumis sativus*	*In vitro* application of CFs	Increases shoot and root lengths and plant dry and fresh weight	CFs contain GAs	Hamayun et al. (2010)
***Penicillium minioluteum* LHL09**	Beneficial	Czapek’s broth	*Oryza sativa (waito-C)/Glycine max*	*In vitro* application of CFs	Promotes plant growth and nitrogen assimilation, with and without sodium chloride-induced salinity	CFs contain GAs	Khan et al. (2011)
***Piriformospora indica***	Beneficial	*Aspergillus*minimal medium	*Helianthus annus L*	Root irrigation with CFs	Increases plant growth and seed production. Increases oil content in seeds	Unknown	Bagde et al. (2011)
***Shimizuomyces paradoxus***	Pathogenic	PDB	*Brassica napus*	Pretreatment of seeds with CF and foliar spray	Increases seed germination and seedling growth	Unknown	Sung et al. (2011)
***Penicillium citrinum* KACC43900**	Beneficial	Czapek’s broth	*Carex kobomugi*	Injection/foliar spray with CFs	Increases leaf blade length, chlorophyll and carotenoids contents and photosynthesis	Unknown	Hwang et al. (2011)
***Fusarium oxysporum***	Pathogenic	CYA	*Cajanus cajan*	Pretreatment of seeds with CF	Increases seed germination and promotes plant growth	Unknown	Jalander and Gachande (2012)
***Penicillium* sp.**	Beneficial	Czapek’s broth	*Suaeda japonica*	*In vitro* application of CFs	Increases plant lenght	CFs contain GAs	You (2012)
***Trichoderma virens***	Beneficial	Richard’s solution	*Capsicum annuum*	Seed coating with CFs	Enhance seed germination and plant vigor	Unknown	Rahman et al. (2012)
***Trichoderma pseudokoningii***							
***Trichoderma harzianum***							
***Penicillium* sp. PNF2**	Beneficial	PDB	*Sesamum indicum*	Pretreatment of seeds with CFs	Increases shoot length and fresh weight	CFs contain IAA	Radhakrishnan et al. (2013)
***Fusarium oxysporum***	Pathogenic	LGN	*Solanum lycopersicum*	Pretreatment of seeds with CFs	Inhibit seed germination and plant growth	CFs contain toxins	Raithak and Gachande (2013)
***Alternaria solani***							
***Penicillium* spp.**	Beneficial/ Pathogenic	ME	*Triticum aestivum*	Pretreatment of seeds with CFs	Increases seed germination and plant biomass	Unknown	Khokhar et al. (2013)
***Piriformospora indica***	Beneficial	*Aspergillus*minimal medium	*Aristolochia elegans*	Soil irrigation with CFs	Increases shoot and root length and fresh and dry weight	Unknown	Bagde et al. (2013)
***Trichoderma* spp.**	Beneficial	ME	*Cicer arietinum*	Pretreatment of seeds with CFs	Increases seed germination and promote plant growth	Unknown	Ali et al. (2014)
***Penicillium* spp. (*NICS01, DFC01*)**	Beneficial	PDB	*Sesamum indicum*	Pretreatment of seeds with CFs	Increase shoot and root length and fresh and dry weight	Amino acids	Radhakrishnan et al., 2014
***Pseudomonas* sp.**	Beneficial	King´s B medium	*Coriandrum sativum*	Pretreatment of seeds with CF and foliar spray	Increases fresh and dry weight and oil production	Unknown	Hegazi et al. (2015)
***Alternaria alternata***	Pathogenic	Czapek’s broth	*Triticum aestivum*	Pretreatment of seeds with CF and foliar spray	Increase seed germination and plant growth	Unknown	Bhajbhuje (2015)
***Alternaria solani***							
***Penicillium nordicum***	Pathogenic	CYA	*Sorghum bicolor*	Pretreatment of seeds with CFs	Inhibit seed germination	CFs contain mycotoxin	Vankudoth et al. (2015)
***Penicillium citrinum*,**							
***Penicillium chrysogenum***							
***Penicillium commune***							
***Penicillium verrucosum***							
***Penicillium camemberti***							
***Penicillium digitatum***							
***Penicillium oxalicum***	Beneficial	PDB	*Pennisetum glaucum*	Pretreatment of seeds with CFs	Increases seed germination and seedling vigor	Unknown	Murali and Amruthesh (2015)
***Fusarium tricinctum RSF-4L***	Pathogenic	Czapek’s broth	*Oryza sativa L.* cv. *Dongjin*	*In vitro* application of CFs	Increase shoot and root lengths, plant fresh weight, and chlorophyll content	CFs contain IAA	Khan et al. (2015)
***Alternaria alternata RSF-6L***							
***Purpureocillium lilacinum***	Beneficial	Specific medium	*Solanum lycopersicum*	Pretreatment of seeds with CFs/Soil irrigation with CFs	Increases seed germination and promotes plant growth	CFs contain IAA	Cavello et al. (2015)
***Fusarium oxysporum***	Pathogenic	Czapek’s broth	*Triticum aestivum* *Hordeum vulgare* *Solanum tuberosum*	Pretreatment of seeds with CFs	Inhibit seed germination and plant growth	Unknown	Ogórek (2016)
***Fusarium sulphureum***							
***Gibberella avenacea***							
***Gibberella intrincans***							
***Trichoderma harzianum* WKY1**	Beneficial	Czapek’s broth	*Shorgum*	Soil irrigation with CFs	Increase shoot and root lengths, plant fresh weight, and total phenol content	CFs contain IAA	Saber et al. (2017)
***Aspergillus fumigatus* TS1**	Beneficial	Czapek’s broth	*Oryza sativa* *(waito-C)*	Application to the apical meristem	Enhance chlorophyll content, root-shoot length, and biomass production	CFs contain IAA and GAs	Bilal et al. (2018)
***Fusarium proliferatum BRL1***							
***Fusarium solani***	Pathogenic	Richard’s solution	*Solanum lycopersicum* *Brassica rapa* *Raphanus sativus* *Trigonella melongena*	Pretreatment of seeds with CFs	Enhances seed germination	Unknown	Parveen et al. (2019)
***Trichothecium roseum***					Inhibit seed germination	CFs contain mycotoxins	
***Aspergillus niger***							
***Cladoporium herbarum***							
***Alternaria alternata***							
***Penicillium chrysogenum***							
***Penicillium expansum***							
***Trichoderma* spp.**	Beneficial				Enhance seed germination	Unknown	
***Trichoderma asperellum***							
***Trichoderma harzianum***							
***Piriformospora indica***	Beneficial	CM	*Cichorium intybus*	Foliar spray	Enhances growth and morpho-physiological traits	Unknown	Rashnoo et al. (2020)
***Trichoderma harzianum*,**	Beneficial	MS	*Capsicum annuum*	Soil irrigation with CFs and DEs	Stimulate root growth and enhance fruit yield	CFs produce changes in plant-associated microbiota	Baroja-Fernández et al. (2021)
***Alternaria alternata***	Pathogenic						
***Penicilium aurantiogriseum***							
***Gibberella intermedia***	Beneficial	Czapek’s broth	*Oryza sativa (waito-C)*	Application to the apical meristem	Increases shoot growth	CFs contain GAs	Khalmuratova et al. (2021)
***Fusarium oxysporum***	Pathogenic	PDB	*Arabidopsis thaliana*	Soil irrigation with CFs	Increase shoot and root biomass but not production	Unkwnon	Ávila and Poveda (2022)
***Pythium irregulare***							
***Rhizoctonia solani***							
***Chaetomium globosum***	Beneficial	ME	*Cichorium intybus*	Soil irrigation with CFs	Increase of biomass, shoots and roots length, and leaf area	Increases phenylalanine pathway and chicoric acid	Spinelli et al. (2022)
***Minimedusa polyspora***						Increases phenylalanine pathway and 4-OH-benzoate	
***Geotrichum candidum***	Neutral	PDB	*Vigna radiata*	Pretreatment of seeds with CFs	Increases seed germination and promotes plant growth	CFs contain IAA	George et al. (2019)
***Saccharomyces cerevisiae***	Beneficial	Sucrose	*Coreandrum sativum*	Pretreatment of seeds with CF and foliar spray	Increases fresh and dry weight and oil production	Unknown	Hegazi et al. (2015)

IAA, Indole-3-acetic acid; GAs, Gibberellins; AMS, Ammonium mineral salt medium supplemented with methanol; CM, Complex medium; CYA, Czapek yeast medium; GNB, Glucose-enriched nutrient broth; GYMA broth, Glucose, yeast extract, malt extract; LB, Luria-Bertani broth; LGN, Liquid glucose nitrate medium; MBGM, Modified bouillon glycerol medium; ME, Malt extract broth; MS, Murashige and Skoog medium; MYP broth, Malta Yeast Peptone broth; PDB, Potato Dextrose Broth; NFb, Nitrogen-free malate; NFDM, Nitrogen-free dextrose; SBM, Sporulation Bacillus Medium; TSB, Trypticase Soy Broth; TYB, Tryptone-Yeast Extract Broth. Specific medium, see publication for details.

**Figure 1:**
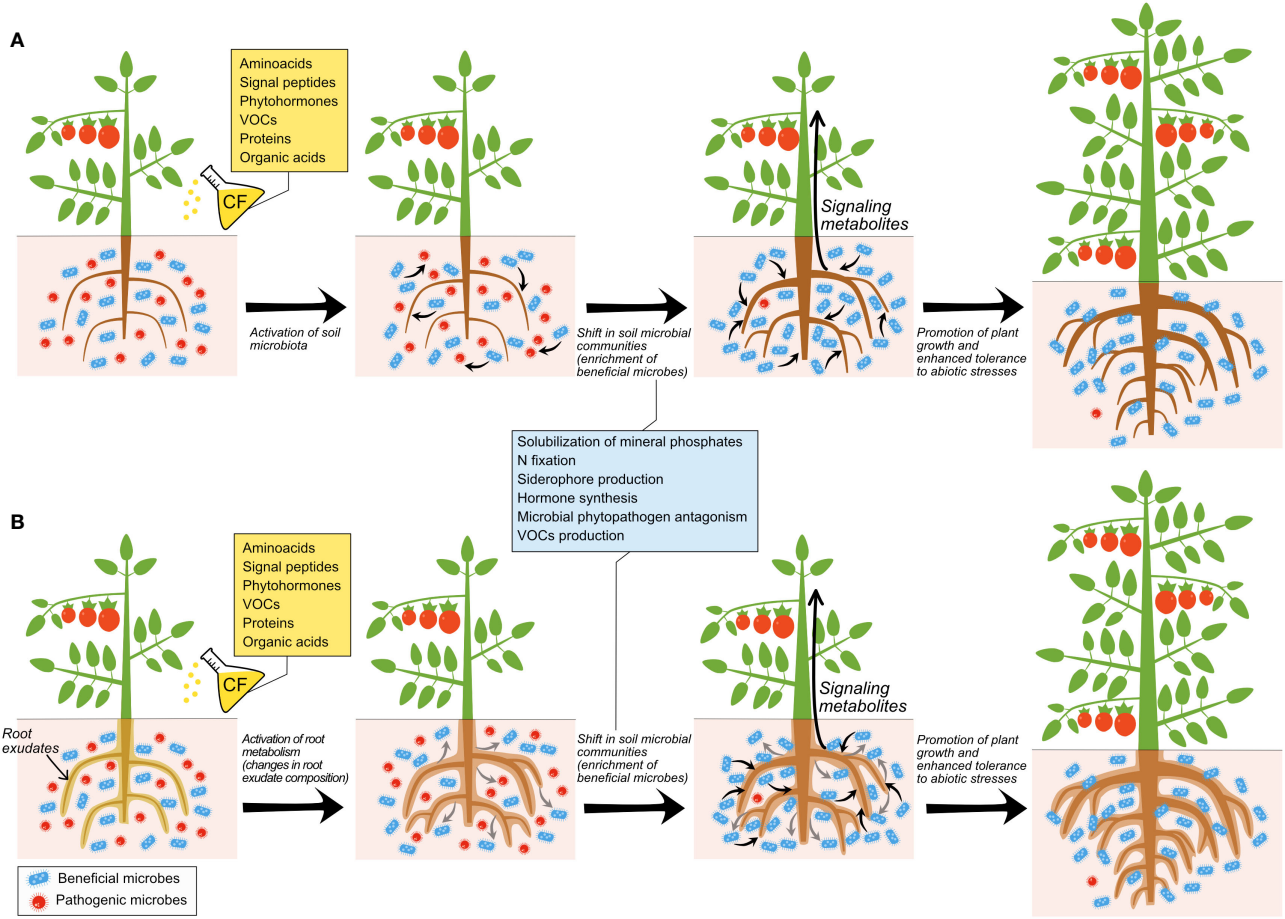
Different scenarios of responses of plants and microbiota to soil application of cell-free microbial CFs. **(A)** illustrates direct action of microbial CF compounds on roots and/or on beneficial microorganisms, which release compounds that exert antagonistic effects on pathogenic microorganisms and/or promote plant growth. **(B)** illustrates direct action of microbial CF compounds on the root exudate composition, which modulates rhizosphere microbiota by impovershing populations of pathogenic microorganisms and enriching those of beneficial microorganisms, which in turn promote plant growth and enhance the capacity of plants to adapt to their environment.

Baroja-Fernández et al. (2021) have recently shown that soil application of CFs of beneficial and phytopathogenic fungi cultured in Murashige & Skoog (MS) medium promoted root growth, enhanced fruit yield and altered composition of fruits of pepper plants. In the same study, the authors found that CFs of the different fungal species possessed volatile organic compounds (VOCs) that, once distilled and applied to soil, promoted responses similar to those triggered by direct application of the fungal CFs. These findings indicated that (i) CFs of both beneficial and phytopathogenic fungi can be used to improve crop yield and (ii) VOCs mediate the crops’ responses to fungal CF application. Some bioactive VOCs present in the fungal CFs are shown in **Table 3**. As further discussed below, it is conceivable that some these compounds (particularly acetic acid) are involved in the crop response to soil application of CFs. Notably, high-throughput sequencing analyses revealed that soil application of fungal CFs and distillates (DEs) promoted similar changes in the soil microbiota, and promoted the proliferation of the same beneficial microbial taxa (Baroja-Fernández et al., 2021) (**Table 4**). Collectively, the findings of Baroja-Fernández et al. (2021) indicated that (i) CFs of both beneficial and fungal phytopathogens can be used to activate the soil and plant-associated beneficial microbiota, and (ii) microbial VOCs mediate the plants’ responses to soil application of fungal CFs through mechanisms involving stimulation of the beneficial soil microbiota as schematically illustrated in **Figure 1**.

**Table 3:** List of potentially relevant bioactive VOCs present in at least one of the fungal CFs used by Baroja-Fernández et al. (2021) and their effects on plant.

VOCs	Effect on plant	Reference
**1-butanol-3-methyl**	Enhances fresh shoot and root biomass and chlorophyll content in Arabidopsis and increases both root length and thickness in *Agave salmiana*.	Camarena-Pozos et al. (2019)
	Increases size, fresh weight and total chlorophyll content in Arabidopsis	Lee et al. (2019a)
**1-butanol-2-methyl**	Increases size, fresh weight and total chlorophyll content in Arabidopsis	Lee et al. (2019a)
**1-hexanol**	Promotes growth of Arabidopsis	Blom et al. (2011)
**benzaldehyde**	Increases the volatile oil accumulation in *Atractylodes lancea*	Zhou et al. (2016)
**2-phenylethyl alcohol**	Enhances fresh shoot and root biomass, chlorophyll content, in Arabidopsis and increases both root length and thickness in *A*. *salmiana*	Camarena-Pozos et al. (2019)
**acetic acid**	Enhances drought tolerance in Arabidopsis, rapeseed, maize, rice and wheat plants	Kim et al. (2017)
	Increases root biomass and photosynthesis in mung bean	Rahman et al. (2019)
**acetoin**	Increases root length, shoot length and dry weight in *L. sativa* seedlings	Fincheira et al. (2017)
	Induces stomatal closure in Arabidopsis and *Nicotiana benthamiana*	Wu et al. (2018)
**2,3-butanediol**	Promotes growth of Arabidopsis	Ryu et al. (2003)
	Induces stomatal closure in Arabidopsis and *N. benthamiana*	Wu et al. (2018)
	Increases root length, shoot length and dry weight in *L. sativa* seedlings	Fincheira et al. (2017)
**2-heptanone**	Promotes the growth of Arabidopsis seedlings	Jiang et al. (2019)
**2-nonanone**	Increases root length, shoot length and dry weight in *L. sativa* seedlings	Fincheira et al. (2017)
**acetophenone**	Elicits the increase of biomass in Arabidopsis	Camarena-Pozos et al. (2019)
**cis-thujopsene**	Induces lateral root formation of Arabidopsis seedlings and poplar plantlets	Ditengou et al. (2015)

**Table 4:** List of microbial species whose populations are enriched by the soil application of fungal CFs and DEs used by Baroja-Fernández et al. (2021).

Microbial species	Mechanism/mode of action	Reference
Bacterial species		
***Burkholderia arboris***	Solubilizes phosphate and produces IAA and siderophores	Zhang et al. (2022)
***Burkholderia silvatlantica***	Has ACC deaminase activity Fixes N_2_	Onofre-Lemus et al. (2009)
		Perin et al. (2006)
***Caballeronia udeis***	Solubilizes phosphate and produces siderophores	Puri et al. (2020)
***Duganella ginsengisoli***	Produces IAA	Goodwin (2022)
***Pseudomonas brassicacearum***	Has ACC deaminase activity	Belimov et al. (2007)
***Pseudomonas mediterranea***	Solubilizes organic phosphate and produces siderophores, proteases, ammonia and IAA	Gu et al. (2020)
***Pseudomonas Knackmussii***	Solubilizes phosphate and produces IAA and siderophores	Rabhi et al. (2018)
***Rhodanobacter glycinis***	Synthesizes osmolytes and biocontrol-related substances	Lee et al. (2019b)
Fungal species		
***Candida subhashii***	Biological control of plant pathogenic fungi	Hilber-Bodmer et al. (2017)
***Geotrichum candidum***	Produces phytohormones and reactive oxygen species	Waqas et al. (2017)
	Solubilizes phosphate	Wu et al. (2012)
	Produces IAA, ammonia and polyamines	Fu et al. (2016)
	Produces IAA and siderophores and has ACC deaminase activity	George et al. (2019)
***Pseudogymnoascus* spp.**	Solubilizes phosphate	Abdel-Ghany et al. (2019)

## Challenges and limitations of the microbial CF technology

**Figure 2:**
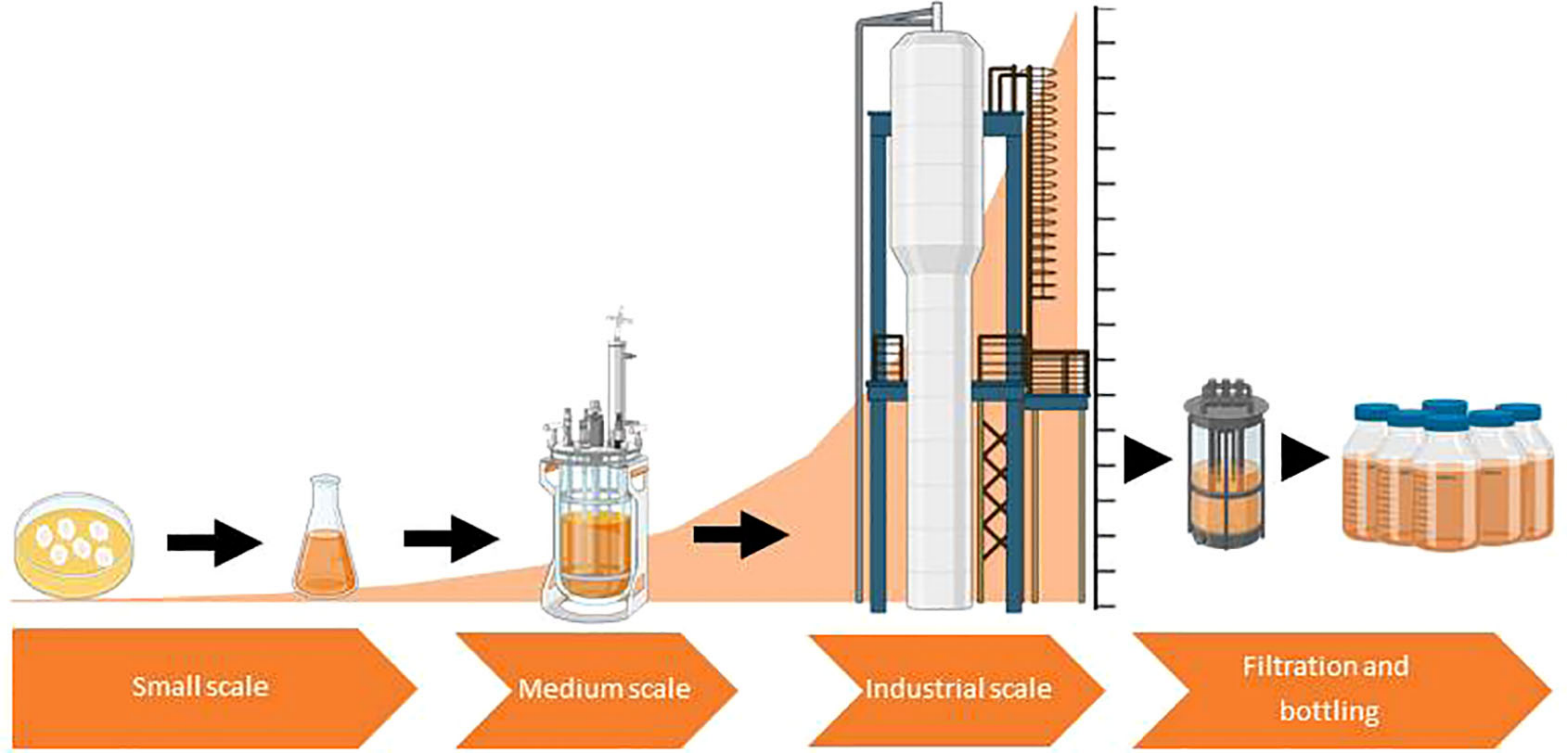
Scheme illustrating the scaling-up process of cell-free microbial culture filtrates (CFs) production to industrial level. Figure was created using BioRender.com.

Despite having great potential as a strategy for improving productivity in a sustainable and eco-friendly manner, the technology based on microbial CF application is still at its infancy and faces important challenges and limitations before it can be widely used (**Table 1**). First, one challenging aspect of the microbial CF-based technology is the manner of application of the extracts, especially in cases in which bioactivity of CFs is based on compounds with high evaporation rates such as VOCs. Most studies on the effect of application of microbial CFs on plants are based on seed coating and soil applications of CFs (**Table 2**), but other means of delivery, should be explored to develop appropriate and durable methods that can be used in the field. Second, another challenging aspect of the microbial CF-based technology is the scaling up from lab-scale shake flasks to stirred tanks-based pilot-scale production (**Figure 2**). This also applies to the technologies of soil inoculation of beneficial microorganisms and application of microbial-derived compounds. Overcoming this limitation is not an easy task due to marked differences in hydromechanical properties and nutrients/oxygen gradients between flasks and large fermentors. For successful scaling up, key parameters affecting heat, momentum and mass transfer should be considered (Trujillo-Roldán et al., 2013). Moreover, some physical parameters should be combined to obtain dimensionless numbers intended to be kept constant during the scaling up process. Third, there are no studies on techno-economic viability of large-scale production of microbial CFs involving (1) propagation of the microbial strain until desired inoculum concentration is reached, (2) fermentation of the microbial strains in large, industrial size fermentors until desired cell concentration is reached and (3) microbial cell removal. For the first two steps, some techno-economic models have been developed for typical liquid biofertilizer production plants (Pérez-Sánchez et al., 2018). These models can be used to investigate the main factors that affect the production process, in order to optimize plant productivity and reliability, and also to reduce costs. Fourth, the limitations to microbial CF use are closely related to the downstream processes for production. However, there are no studies on the formulation and shelf life of microbial CFs and on allocation of fitness costs for resources for the large-scale synthesis of these extracts. Clearly, the formulation of new products ready to be commercialized requires further scientific and industrial up-scaling studies. Fifth, the effects of CFs on plants and associated microbiota may vary depending on the plant and microbial species and ecotypes as well as on media composition, age and growth conditions of the microbial culture. Thus, whereas Khokhar et al. (2013) reported that application of CFs of several *Penicillium* spp. cultured for 15 days at 20 °C in malt extract broth exerted a positive effect on wheat germination and growth, Vankudoth et al. (2015) reported that application of CFs of the same fungal species cultured for 12 days at 27 °C in CYA broth exerted a negative effect on sorghum germination. CFs of the phytopathogen *Fusarium oxysporum* grown in Czapek-Dox and potato dextrose liquid media exerted a negative effect on seed germination and growth of cucumber and garden cress (*Lepidium sativum* L.) plants (Melo and Piccinin, 1999; Ogórek, 2016), whereas application of CFs of *F. oxysporum* cultured for 5 days at 25 °C in CYA broth enhanced pigeonpea (*Cajanus cajan* L.) seed germination and growth (Jalander and Gachande, 2012). Application of CFs of the fungal phytopathogen *A. alternata* cultured in Czapek broth and MS media promoted growth of rice and wheat plants and enhanced pepper fruit yield (Bhajbhuje, 2015; Khan et al., 2015; Baroja-Fernández et al., 2021), whereas application of CFs of the same species cultured in Richard´s solution exerted a negative effect on germination of seeds of several crop plants (Parveen et al., 2019). In many instances, the growth inhibitory effect of the CFs of phytopathogens was due to toxins released by the microorganism to the culture medium (Raithak and Gachande, 2013; Vankudoth et al., 2015; Parveen et al., 2019). Sixth, above threshold levels, many microbial compounds are toxic to plants. Therefore, excess application of microbial CFs has the potential to exert a negative effect on plants. However, after due assessment of the dose-response effect on specific crops, microbial CFs can be safely managed. Seventh, although Baroja-Fernández et al. (2021) showed that application of CFs of diverse microorganisms resulted in activation of beneficial soil and plant-associated microbiota without significant changes in the relative abundance of populations of pathogenic microbial species, it is important to ensure that these results can be extrapolated to other CFs in different soil types and environmental scenarios. Eighth, CFs based on co-cultivation of various microorganisms might be an efficient approach to obtain widely range of bioactive compounds. Nevertheless, this practice faces similar problems to those of multi-microbial bioinoculants, since each co-inoculant requires specific culture conditions (Reddy and Saravanan, 2013). Nineth, there are few studies on the mechanisms and modes of action of microbial CFs on plants.

## The necessity of identifying the mechanisms and modes of action of cell-free microbial CFs for their use as biostimulants

In our opinion, the exploitation of microbial CFs as biostimulants (defined by the European Biostimulant Industry Council (https://biostimulants.eu/) as “*substances and/or microorganisms whose function when applied to plants or to soil is to stimulate natural processes to enhance or benefit nutrient uptake, nutrient efficiency, tolerance to abiotic stress and crop quality*” (du Jardin, 2015)) is only just evolving and its broad potential is now beginning to be demonstrated. The majority of studies describing the positive effect(s) of the application of microbial CFs on plant growth and yield did not identify modes or mechanisms of action of these extracts (**Table 1**). At most, some of these studies proposed that phytohormones and other growth promoting compounds released by microbes in their culture media are involved in the response of plants to microbial CFs. This may result in the assumption by many that these complex, multicomponent mixtures are “magic potions” or “snake oil” not compliant with scientific requirements of the official regulations for fertilizer products (Yakhin et al., 2017). The identification of mechanisms and modes of action of microbial CFs on plants and the characterization of the biological functions and ecological roles of their bioactive components, based on reasonable scientific hypotheses rather than a try-it-and-see approach, could be important not only to develop novel products able to increase yields in crops in a sustainable and environmentally benign manner, but also to obtain clues for the biotechnological design of more productive and efficient crops.

Baroja-Fernández et al. (2021) showed that VOCs are important mediators in the response of plants and plant-associated microbiota to soil application of microbial CFs. However, the bioactive VOCs produced by fungal cultures and their mechanisms and modes of action remain unverified. Furthermore, whether the effect exerted by soil application of VOCs on plants is direct or mediated by changes induced in the composition of the soil microbiota (or both) still needs to be determined, as does whether the effect exerted by soil application of VOCs on the plant-associated microbiota is direct or mediated by changes induced in the root metabolism (**Figure 1**). That VOCs isolated from CFs of diverse beneficial and phytopathogenic microorganisms promoted similar growth and fruit yield and quality responses in crops indicate that plants respond similarly to a wide range of bioactive microbial VOCs. Alternatively, it is likely that many microorganisms produce the same growth promoting VOCs. One of such compounds could be acetic acid, which is present in soils, where microorganisms produce it in response to biotic and abiotic stresses (Adeleke et al., 2017). Recent studies have shown that soil application of acetic acid, but not other organic acids, enhances drought tolerance in Arabidopsis and important crops including maize, wheat, rice and cassava (Kim et al., 2017; Utsumi et al., 2019) and tolerance to bacterial wilt disease (Wang et al., 2021). In Arabidopsis, the enhancement of drought resistance promoted by soil application of acetic acid involves JA signaling and an ON/OFF switching epigenetic mechanism dependent on histone deacetylase HDA6 (Kim et al., 2017). However, the biochemical and molecular mechanisms underlying the enhancement of drought tolerance promoted by soil application of acetic acid in crops remain to be elucidated.

Several lines of evidence indicate that the manner of application of microbial volatile compounds and/or plant growth conditions are important determinants of the biochemical and molecular mechanisms triggered in plants. Application of these compounds *via* the air promotes plant growth and metabolic and developmental changes, enhances photosynthesis and improves nutrient and water acquisition (Ryu et al., 2003; Zhang et al., 2008; Zhang et al., 2009; Ezquer et al., 2010; Gutiérrez-Luna et al, 2010; Garnica-Vergara et al., 2016; Sánchez-López et al., 2016; García-Gómez et al., 2019; García-Gómez et al., 2020). In *Arabidopsis*, these responses are associated with changes in the transcriptome, proteome, metabolome, hormonome and redox-proteome through mechanisms involving long-distance communication between roots and the aerial part of the plant and proteostatic regulation of central metabolic pathways in the plastidial compartment (Zhang et al., 2007; Zhang et al., 2008; Zhang et al., 2009; Sánchez-López et al., 2016; Ameztoy et al., 2019; García-Gómez et al., 2020; Ameztoy et al., 2021; Gámez-Arcas et al., 2022). However, Baroja-Fernández et al. (2021) showed that no such mechanisms operate in crops irrigated with VOC-containing microbial CFs and DEs. Needless to say, further work will be necessary to understand how plants respond to microbial volatiles.

The discovery that soil application of VOCs from diverse microorganisms can enhance crop yield and promote similar changes in the soil microbiota extends knowledge on the mechanisms modulating the physiology of the plant and their interaction with plant-associated microbes, raising questions on their ecological significance and biotechnological applications. Because microbes respond to microbial VOCs, it is likely that the fungal VOC-promoted enrichment of the soil beneficial microbiota is due to direct action of these compounds on the microorganisms, which in turn determine plant growth and metabolism through emission of semiochemicals. These compounds trigger the production of molecules by roots that, once transported to the aerial part of the plant, act as long-distance signals that promote growth and enhance yield (**Figure 1A**). In line with this presumption, Yuan et al. (2017) showed that exposure of soil to VOCs of *B. amyloliquefaciens* NJN-6 altered the composition of soil microbial communities. Compounds secreted by roots in their exudates attract beneficial microorganisms which, in turn, strongly influence plant fitness and enhance the capacity of plants to adapt to environmental changes and stress (Baudoin and Benizri, 2003; Badri and Vivanco, 2009; Badri et al., 2013; Bulgarelli et al., 2013; Schulz-Bohm et al., 2017; Sasse et al., 2018; Zhalnina et al., 2018; Huang et al., 2019; Chen et al., 2020; Vílchez et al., 2020; He et al., 2022). Therefore, it is conceivable that enrichment in the plant-associated beneficial microbiota triggered by application of fungal CFs and DEs is due to an alteration of composition of root exudates (**Figure 1B**). This capacity of root exudates to influence the soil microbiota is not static since the composition of root exudates is not uniform over time (Zhalnina et al., 2018) and depends on the plant species or genotype (Bulgarelli et al., 2012; Bodenhausen et al., 2014; Bouffaud et al., 2014; Zhong et al., 2019). For instance, it has been shown that soybean genotype significantly influences the structure and composition of its associated rhizosphere and affects connections in rhizobacterial networks (Zhong et al., 2019).

## Additional remarks

According to the Regulation (EU) 2019/1009 of the European Parliament and of the Council of 5 June 2019 laying down rules on the making available on the market of EU fertilising products, a microbial plant biostimulant may contain micro-organisms belonging to the *Azotobacter Rhizobium*, *Azospirillum* genera and mycorrhizal fungi, including dead or empty-cell micro-organisms and non-harmful residual elements of the media on which the microorganisms were cultured (https://eur-lex.europa.eu/legal-content/EN/TXT/PDF/?uri=CELEX:32019R1009). Although regulations are of great importance for guaranteeing food security and preserving environmental integrity, the stringency and exclusivity of the list of microorganisms that can be used for the production of biostimulants may strongly limit the potential benefits of these products. As shown in the review, growing evidence has been compiled demonstrating that application of cell-free CFs of beneficial and phytopathogenic microorganisms is an efficient approach to promote plant growth and enhance yield and stress tolerance in a wide range of crops while reducing the use of agrochemicals. Therefore, it may be appropriate to enlarge the list of microorganisms that can be used for the production of cell-free CF-based biostimulants according to EU regulation on fertilising products, assuming scientific evidence can demonstrate that these products are safe for both the environment and consumers.

## Authors contributions

RL, EB-F and J-PR conceptualized the topic and wrote the manuscript. All authors contributed in the literature search and discussions, and reviewed and approved the final manuscript.

## References

[B1] Abdel-Ghany T. M. Mohamed Z. H. Al Abboud M. A. Helmy E. A. Al-Rajhi A. M. H. Shater A. R. M. (2019). Solubilization of inorganic phosphate by rhizospheric fungi isolated from soil cultivated with *Sorghum bicolor* l. BioRes. 14 (3), 5521–5532. doi: 10.15376/biores.14.3.5521-5532

[B2] Adeleke R. Nwangburuka C. Oboirien B. (2017). Origins, roles and fate of organic acids in soils: A review. S. Afr. J. Bot. 108, 393–406. doi: 10.1016/J.SAJB.2016.09.002

[B3] Ahmad M. Pataczek L. Hilger T. H. Zahir Z. A. Hussain A. Rasche F. . (2018). Perspectives of microbial inoculation for sustainable development and environmental management. Front. Microbiol. 9. doi: 10.3389/FMICB.2018.02992 PMC6289982 30568644

[B4] Aldesuquy H. S. Mansour F. A. Abo-Hamed S. A. (1998). Effect of the culture filtrates of *Streptomyces* on growth and productivity of wheat plants. Folia Microbiol. 43 (5), 465–470. doi: 10.1007/BF02820792

[B5] Ali A. Haider M. S. Ashfaq M. Hanif S. (2014). Effect of culture filtrates of *Trichoderma* spp. on seed germination and seedling growth in chickpea–an *in-vitro* study. Pakistan J. Phytopathol. 26 (1), 01–05.

[B6] Ameztoy K. Baslam M. Sánchez-López Á.M. Muñoz F. J. Bahaji A. Almagro G. . (2019). Plant responses to fungal volatiles involve global posttranslational thiol redox proteome changes that affect photosynthesis. Plant Cell Environ. 42 (9), 2627–2644. doi: 10.1111/pce.13601 31222760

[B7] Ameztoy K. Sánchez-López Á.M. Muñoz F. J. Bahaji A. Almagro G. Baroja-Fernández E. . (2021). Proteostatic regulation of MEP and shikimate pathways by redox-activated photosynthesis signaling in plants exposed to small fungal volatiles. Front. Plant Sci. 12. doi: 10.3389/fpls.2021.637976 PMC7973468 33747018

[B8] Arkhipova T. N. Veselov S. U. Melentiev A. I. Martynenko E. V. Kudoyarova G. R. (2005). Ability of bacterium *Bacillus subtilis* to produce cytokinins and to influence the growth and endogenous hormone content of lettuce plants. Plant Soil. 272 (1), 201–209. doi: 10.1007/s11104-004-5047-x

[B9] Ávila A. C. Poveda J. (2022). Induction of immune response in *Arabidopsis thaliana* treated with phytopathogen filtrates. Biol. Life Sci. Forum. 11, 85. doi: 10.3390/IECPS2021-11974

[B10] Azcón R. Barea J. M. (1975). Synthesis of auxins, gibberellins and cytokinins by *Azotobacter vinelandii* and *Azotobacter beijerinckii* related to effects produced on tomato plants. Plant Soil. 43 (1), 609–619. doi: 10.1007/BF01928522

[B11] Backer R. Rokem J. S. Ilangumaran G. Lamont J. Praslickova D. Ricci E. . (2018). Plant growth-promoting rhizobacteria: Context, mechanisms of action, and roadmap to commercialization of biostimulants for sustainable agriculture. Front. Plant Sci. 871. doi: 10.3389/FPLS.2018.01473 PMC6206271 30405652

[B12] Badri D. V. Chaparro J. M. Zhang R. Shen Q. Vivanco J. M. (2013). Application of natural blends of phytochemicals derived from the root exudates of arabidopsis to the soil reveal that phenolic-related compounds predominantly modulate the soil microbiome. J. Biol. Chem. 288 (7), 4502–4512. doi: 10.1074/JBC.M112.433300 23293028 PMC3576057

[B13] Badri D. V. Vivanco J. M. (2009). Regulation and function of root exudates. Plant Cell Environ. 32 (6), 666–681. doi: 10.1111/j.1365-3040.2009.01926.x 19143988

[B14] Bagde U. Prasad R. Varma A. (2011). Influence of culture filtrate of *Piriformospora indica* on growth and yield of seed oil in *Helianthus annus* . Symbiosis. 53, 83–88. doi: 10.1007/s13199-011-0114-6

[B15] Bagde U. S. Prasad R. Varma A. (2013). Impact of culture filtrate of *Piriformospora indica* on biomass and biosynthesis of active ingredient aristolochic acid in *Aristolochia elegans* . Mart. Int. J. Biol. 6, 29. doi: 10.5539/ijb.v6n1p29

[B16] Baroja-Fernández E. Almagro G. Sánchez-López Á.M. Bahaji A. Gámez-Arcas S. De Diego N. . (2021). Enhanced yield of pepper plants promoted by soil application of volatiles from cell-free fungal culture filtrates is associated with activation of the beneficial soil microbiota. Front. Plant Sci. 12. doi: 10.3389/fpls.2021.752653 PMC8566893 34745186

[B17] Bashan Y. de-Bashan L. E. Prabhu S. R. Hernandez J.-P. (2014). Advances in plant growth-promoting bacterial inoculant technology: formulations and practical perspective–2013). Plant Soil. 378 (1), 1–33. doi: 10.1007/s11104-013-1956-x

[B18] Baudoin E. Benizri E. (2003). Impact of artificial root exudates on the bacterial community structure in bulk soil and maize rhizosphere. Soil Biol. Biochem. 35, 1183–1192. doi: 10.1016/S0038-0717(03)00179-2

[B19] Belimov A. A. Dodd I. C. Safronova V. I. Hontzeas N. Davies W. J. (2007). *Pseudomonas brassicacearum* strain Am3 containing 1-aminocyclopropane-1-carboxylate deaminase can show both pathogenic and growth-promoting properties in its interaction with tomato. J. Exp. Bot. 58 (6), 1485–1495. doi: 10.1093/JXB/ERM010 17322547

[B20] Berg G. (2009). Plant-microbe interactions promoting plant growth and health: perspectives for controlled use of microorganisms in agriculture. Appl. Microbiol. Biotechnol. 84 (1), 11–18. doi: 10.1007/s00253-009-2092-7 19568745

[B21] Berg G. Kusstatscher P. Abdelfattah A. Cernava T. Smalla K. (2021). Microbiome modulation–toward a better understanding of plant microbiome response to microbial inoculants. Front. Microbiol. 12. doi: 10.3389/fmicb.2021.650610 PMC8060476 33897663

[B22] Bhajbhuje M. N. (2015). Response of metabolites from culture filtrates of *Alternaria* species against *Triticum aestivum* l. Int. J. Life Sci. 3, 55–62.

[B23] Bilal L. Asaf S. Hamayun M. Gul H. Iqbal A. Ullah I. . (2018). Plant growth promoting endophytic fungi *Aspergillus fumigatus* TS1 and *Fusarium proliferatum* BRL1 produce gibberellins and regulates plant endogenous hormones. Symbiosis. 76, 117–127. doi: 10.1007/s13199-018-0545-4

[B24] Blom D. Fabbri C. Connor E. C. Schiestl F. P. Klauser D. R. Boller T. . (2011). Production of plant growth modulating volatiles is widespread among rhizosphere bacteria and strongly depends on culture conditions. Environ. Microbiol. 13 (11), 3047–3058. doi: 10.1111/j.1462-2920.2011.02582.x 21933319

[B25] Bodenhausen N. Bortfeld-Miller M. Ackermann M. Vorholt J. A. (2014). A synthetic community approach reveals plant genotypes affecting the phyllosphere microbiota. PloS Genet. 10 (4), e1004283. doi: 10.1371/journal.pgen.1004283 24743269 PMC3990490

[B26] Bouffaud M. L. Poirier M. A. Muller D. Moënne-Loccoz Y. (2014). Root microbiome relates to plant host evolution in maize and other poaceae. Environ. Microbiol. 16 (9), 2804–2814. doi: 10.1111/1462-2920.12442 24588973

[B27] Buensanteai N. Yuen G. Y. Prathuangwong S. (2008). The biocontrol bacterium *Bacillus amyloliquefaciens* KPS46 produces auxin, surfactin and extracellular proteins for enhanced growth of soybean plant. Thai J. Agric. Sci. 41 (3–4), 101–116.

[B28] Buensateai N. Sompong M. Thamnu K. Athinuwat D. Brauman A. Plassard C. (2013). The plant growth promoting bacterium *Bacillus* sp. CaSUT007 produces phytohormone and extracellular proteins for enhanced growth of cassava. Afr. J. Microbiol. Res. 7 (42), 4949–4954. doi: 10.5897/AJMR12.1839

[B29] Bulgarelli D. Rott M. Schlaeppi K. Ver Loren van Themaat E. Ahmadinejad N. Assenza F. . (2012). Revealing structure and assembly cues for arabidopsis root-inhabiting bacterial microbiota. Nature. 488 (7409), 91–95. doi: 10.1038/nature11336 22859207

[B30] Bulgarelli D. Schlaeppi K. Spaepen S. Van Themaat E. V. L. Schulze-Lefert P. (2013). Structure and functions of the bacterial microbiota of plants. Annu. Rev. Plant Biol. 64, 807–838. doi: 10.1146/annurev-arplant-050312-120106 23373698

[B31] Burkle L. A. Belote R. T. (2015). Soil mutualists modify priority effects on plant productivity, diversity, and composition. Appl. Veg. Sci. 18, 332–342. doi: 10.1111/avsc.12149

[B32] Calvo P. Nelson L. Kloepper J. W. (2014). Agricultural uses of plant biostimulants. Plant Soil 383 (1–2), 3–41. doi: 10.1007/S11104-014-2131-8/TABLES/1

[B33] Camarena-Pozos D. A. Flores-Núñez V. M. López M. G. López-Bucio J. Partida-Martínez L. P. (2019). Smells from the desert: Microbial volatiles that affect plant growth and development of native and non-native plant species. Plant Cell Environ. 42 (4), 1368–1380. doi: 10.1111/PCE.13476 30378133

[B34] Cardinale M. Ratering S. Suarez C. Zapata Montoya A. M. Geissler-Plaum R. Schnell S. (2015). Paradox of plant growth promotion potential of rhizobacteria and their actual promotion effect on growth of barley (*Hordeum vulgare* l.) under salt stress. Microbiol. Res. 181, 22–32. doi: 10.1016/j.micres.2015.08.002 26640049

[B35] Carrión V. J. Perez-Jaramillo J. Cordovez V. Tracanna V. de Hollander M. Ruiz-Buck D. . (2019). Pathogen-induced activation of disease-suppressive functions in the endophytic root microbiome. Science. 366 (6465), 606–612. doi: 10.1126/science.aaw9285 31672892

[B36] Castrillo G. Teixeira P. J. P. L. Paredes S. H. Law T. F. de Lorenzo L. Feltcher M. E. . (2017). Root microbiota drive direct integration of phosphate stress and immunity. Nature. 543 (7646), 513–518. doi: 10.1038/nature21417 28297714 PMC5364063

[B37] Cavello I. Crespo J. García S. Zapiola J. Luna M. Cavalitto S. (2015). Plant growth promotion activity of keratinolytic fungi growing on a recalcitrant waste known as “hair waste”. Biotechnol. Res. Int. 2015, 1–10. doi: 10.1155/2015/952921 PMC4677195 26697226

[B38] Chanclud E. Morel J.-B. (2016). Plant hormones: a fungal point of view. Mol. Plant Pathol. 17 (8), 1289–1297. doi: 10.1111/mpp.12393 26950404 PMC6638337

[B39] Chen Y. Bonkowski M. Shen Y. Griffiths B. S. Jiang Y. Wang X. . (2020). Root ethylene mediates rhizosphere microbial community reconstruction when chemically detecting cyanide produced by neighbouring plants. Microbiome. 8 (1), 4. doi: 10.1186/s40168-019-0775-6 31954405 PMC6969408

[B40] Contreras-Cornejo H. A. Maciías-Rodriíguez L. Corteís-Penagos C. Loípez-Bucio J. (2009). Trichoderma virens, a plant beneficial fungus, enhances biomass production and promotes lateral root growth through an auxin-dependent mechanism in arabidopsis. Plant Physiol. 149 (3), 1579–1592. doi: 10.1104/pp.108.130369 19176721 PMC2649400

[B41] Diagne N. Baudoin E. Svistoonoff S. Ouattara C. Diouf D. Kane A. . (2018). Effect of native and allochthonous arbuscular mycorrhizal fungi on *Casuarina equisetifolia* growth and its root bacterial community. Arid. Land Res. Manage. 32 (2), 212–228. doi: 10.1080/15324982.2017.1406413

[B42] Ditengou F. A. Müller A. Rosenkranz M. Felten J. Lasok H. van Doorn M. M. . (2015). Volatile signalling by sesquiterpenes from ectomycorrhizal fungi reprogrammes root architecture. Nat. Commun. 6 (1), 1–9. doi: 10.1038/ncomms7279 PMC4346619 25703994

[B43] du Jardin P. (2015). Plant biostimulants: Definition, concept, main categories and regulation. Sci. Hortic. 196, 3–14. doi: 10.1016/J.SCIENTA.2015.09.021

[B44] Durán P. Thiergart T. Garrido-Oter R. Agler M. Kemen E. Schulze-Lefert P. . (2018). Microbial interkingdom interactions in roots promote arabidopsis survival. Cell. 175 (4), 973–983.e14. doi: 10.1016/j.cell.2018.10.020 30388454 PMC6218654

[B45] Egamberdieva D. Wirth S. J. Alqarawi A. A. Abd-Allah E. F. Hashem A. (2017). Phytohormones and beneficial microbes: Essential components for plants to balance stress and fitness. Front. Microbiol. 8. doi: 10.3389/fmicb.2017.02104 PMC5671593 29163398

[B46] El-Khawas H. Adachi K. (1999). Identification and quantification of auxins in culture media of *Azospirillum* and *Klebsiella* and their effect on rice roots. Biol. Fertil. Soils. 28 (4), 377–381. doi: 10.1007/s003740050507

[B47] El-Shanshoury A. R. (1989). Growth promotion of wheat seedlings by *Streptomyces atroolivaceus* . J. Agron. Crop Sci. 163 (2), 109–114. doi: 10.1111/j.1439-037X.1989.tb00743.x

[B48] Emmanuel O. C. Babalola O. O. (2020). Productivity and quality of horticultural crops through co-inoculation of arbuscular mycorrhizal fungi and plant growth promoting bacteria. Microbiol. Res. 239, 126569. doi: 10.1016/j.micres.2020.126569 32771873

[B49] Ezquer I. Li J. Ovecka M. Baroja-Fernández E. Muñoz F. J. Montero M. . (2010). Microbial volatile emissions promote accumulation of exceptionally high levels of starch in leaves in mono- and dicotyledonous plants. Plant Cell Physiol. 51 (10), 1674–1693. doi: 10.1093/PCP/PCQ126 20739303

[B50] Fincheira P. Parra L. Mutis A. Parada M. Quiroz A. (2017). Volatiles emitted by *Bacillus* sp. BCT9 act as growth modulating agents on *Lactuca sativa* seedlings. Microbiol. Res. 203, 47–56. doi: 10.1016/J.MICRES.2017.06.007 28754207

[B51] Fiorentino N. Ventorino V. Woo S. L. Pepe O. De Rosa A. Gioia L. . (2018). Trichoderma-based biostimulants modulate rhizosphere microbial populations and improve n uptake efficiency, yield and nutritional quality of leafy vegetables. Front. Plant Sci. 9. doi: 10.3389/fpls.2018.00743 PMC5996573 29922317

[B52] Francis I. Holsters M. Vereecke D. (2010). The gram-positive side of plant-microbe interactions. Environ. Microbiol. 12 (1), 1–12. doi: 10.1111/J.1462-2920.2009.01989.X 19624707

[B53] Fu S.-F. Sun P.-F. Lu H.-Y. Wei J.-Y. Xiao H.-S. Fang W.-T. . (2016). Plant growth-promoting traits of yeasts isolated from the phyllosphere and rhizosphere of *Drosera spatulata* Lab. Fungal Biol. 120, 433–448. doi: 10.1016/j.funbio.2015.12.006 26895872

[B54] Gámez-Arcas S. Baroja-Fernández E. García-Gómez P. Muñoz F. J. Almagro G. Bahaji A. . (2022). Action mechanisms of small microbial volatile compounds in plants. J. Exp. Bot. 73 (2), 498–510. doi: 10.1093/jxb/erab463 34687197

[B55] García-Gómez P. Almagro G. Sánchez-López Á.M. Bahaji A. Ameztoy K. Ricarte-Bermejo A. . (2019). Volatile compounds other than CO_2_ emitted by different microorganisms promote distinct posttranscriptionally regulated responses in plants. Plant Cell Environ. 42 (5), 1729–1746. doi: 10.1111/PCE.13490 30480826

[B56] García-Gómez P. Bahaji A. Gámez-Arcas S. Muñoz F. J. Sánchez-López Á.M. Almagro G. . (2020). Volatiles from the fungal phytopathogen *Penicillium aurantiogriseum* modulate root metabolism and architecture through proteome resetting. Plant Cell Environ. 43 (10), 2551–2570. doi: 10.1111/PCE.13817 32515071

[B57] Garnica-Vergara A. Barrera-Ortiz S. Muñoz-Parra E. Raya-González J. Méndez-Bravo A. Macías-Rodríguez L. . (2016). The volatile 6-pentyl-2H-pyran-2-one from *Trichoderma atroviride* regulates *Arabidopsis thaliana* root morphogenesis *via* auxin signaling and ETHYLENE INSENSITIVE 2 functioning. New Phytol. 209 (4), 1496–1512. doi: 10.1111/NPH.13725 26568541

[B58] George T. K. SubaidaBeevi S. Asok A. K. Shaikmoideen J. M. (2019). Plant growth promoting endophytic yeast *Geotrichum candidum* (jx 477426) from roots of *Bruguiera cylindrica* . J. Microbiol. Biotechnol. Food Sci. 9 (2), 267–272. doi: 10.15414/JMBFS.2019.9.2.267-272

[B59] Ghimire S. R. Charlton N. D. Craven K. D. (2009). The mycorrhizal fungus, *Sebacina vermifera*, enhances seed germination and biomass production in switchgrass (*Panicum virgatum* l). Bioenergy Res. 2 (1), 51–58. doi: 10.1007/s12155-009-9033-2

[B60] Glare T. Caradus J. Gelernter W. Jackson T. Keyhani N. Köhl J. . (2012). Have biopesticides come of age? Trends Biotechnol. 30 (5), 250–258. doi: 10.1016/j.tibtech.2012.01.003 22336383

[B61] Goodwin P. H. (2022). The rhizosphere microbiome of ginseng. Microorganisms 10, 1152. doi: 10.3390/microorganisms10061152 35744670 PMC9231392

[B62] Gutiérrez-Luna F. M. López-Bucio J. Altamirano-Hernández J. Valencia-Cantero E. (2010), Reyes De la Cruz, h., and macías-rodríguez, l. plant growth-promoting rhizobacteria modulate root-system architecture in arabidopsis thaliana through volatile organic compound emission. Symbiosis. 51, 75–83. doi: 10.1007/s13199-010-0066-2

[B63] Gu Y. Wang J. Xia Z. Wei H. L. (2020). Characterization of a versatile plant growth-promoting rhizobacterium *Pseudomonas mediterranea* strain S58. Microorganisms. 8 (3), 334. doi: 10.3390/MICROORGANISMS8030334 32120878 PMC7143339

[B64] Hamayun M. Khan S. A. Khan A. L. Rehman G. Kim Y. H. Iqbal I. . (2010). Gibberellin production and plant growth promotion from pure cultures of cladosporium sp. MH-6 isolated from cucumber (*Cucumis sativus* l.). Mycologia. 102 (5), 989–995. doi: 10.3852/09-261 20943499

[B65] Hart M. M. Antunes P. M. Chaudhary V. B. Abbott L. K. (2018). Fungal inoculants in the field: Is the reward greater than the risk? Funct. Ecol. 32 (1), 126–135. doi: 10.1111/1365-2435.12976

[B66] Hartmann A. Rothballer M. Hense B. A. Schröder P. (2014). Bacterial quorum sensing compounds are important modulators of microbe-plant interactions. Front. Plant Sci. 5. doi: 10.3389/FPLS.2014.00131 PMC3986513 24782873

[B67] Hegazi M. A. Metwaly M. M. S. Belal E. B. (2015). Influence of plant growth promoting bacteria (PGPB) on coriander (*Coriandrum sativum* l.) and dill (*Anethum graveolens* l.) plants. J. Plant Production. 6 (2), 205–218. doi: 10.21608/jpp.2015.49299

[B68] He D. Singh S. K. Peng L. Kaushal R. Vílchez J. I. Shao C. . (2022). Flavonoid-attracted aeromonas sp. from the arabidopsis root microbiome enhances plant dehydration resistance. ISME J. 16, 2622–2632. doi: 10.1038/s41396-022-01288-7 35842464 PMC9561528

[B69] Hilber-Bodmer M. Schmid M. Ahrens C. H. Freimoser F. M. (2017). Competition assays and physiological experiments of soil and phyllosphere yeasts identify *Candida subhashii* as a novel antagonist of filamentous fungi. BMC Microbiol. 17, 4. doi: 10.1186/s12866-016-0908-z 28056814 PMC5216558

[B70] Hiruma K. Gerlach N. Sacristán S. Nakano R. T. Hacquard S. Kracher B. . (2016). Root endophyte *Colletotrichum tofieldiae* confers plant fitness benefits that are phosphate status dependent. Cell. 165 (2), 464–474. doi: 10.1016/J.CELL.2016.02.028 26997485 PMC4826447

[B71] Hoeksema J. D. Chaudhary V. B. Gehring C. A. Johnson N. C. Karst J. Koide R. T. . (2010). A meta-analysis of context-dependency in plant response to inoculation with mycorrhizal fungi. Ecol. Lett. 13 (3), 394–407. doi: 10.1111/j.1461-0248.2009.01430.x 20100237

[B72] Huang A. C. Jiang T. Liu Y. X. Bai Y. C. Reed J. Qu B. . (2019). A specialized metabolic network selectively modulates arabidopsis root microbiota. Science. 364 (6440), eaau6389. doi: 10.1126/SCIENCE.AAU6389 31073042

[B73] Hwang J. S. You Y. H. Bae J. J. Khan S. A. Kim J. G. Choo Y. S. (2011). Effects of endophytic fungal secondary metabolites on the growth and physiological response of *Carex kobomugi* ohwi. J. Coast. Res. 27 (3), 544–548. doi: 10.2112/JCOASTRES-D-10-00090.1

[B74] Idris E. E. S. Bochow H. Ross H. Borriss R. (2004). Use of *Bacillus subtilis* as biocontrol agent. VI. phytohormone-like action of culture filtrates prepared from plant-growth *promoting bacillus amyloliquefaciens* FZB24, FZB42, FZB45 and *Bacillus subtilis* FZB37. J. Plant Dis. Prot. 111, 583–597.

[B75] Jalander V. Gachande B. D. (2012). Effect of fungal metabolites of some rhizosphere soil fungi on seed germination and seedling growth of some pulses and cereals. Sci. Res. Repor. 2 (3), 265–267.

[B76] Jiang C. H. Xie Y. S. Zhu K. Wang N. Li Z. J. Yu G. J. . (2019). Volatile organic compounds emitted by bacillus sp. JC03 promote plant growth through the action of auxin and strigolactone. Plant Growth Regul. 87 (2), 317–328. doi: 10.1007/S10725-018-00473-Z

[B77] Kanchiswamy C. N. Malnoy M. Maffei M. E. (2015). Chemical diversity of microbial volatiles and their potential for plant growth and productivity. Front. Plant Sci. 6. doi: 10.3389/FPLS.2015.00151 PMC4358370 25821453

[B78] Kaur T. Rani R. Manhas R. K. (2019). Biocontrol and plant growth promoting potential of phylogenetically new *Streptomyces* sp. MR14 of rhizospheric origin. AMB Express. 9 (1), 125. doi: 10.1186/S13568-019-0849-7 31399889 PMC6689040

[B79] Khalmuratova I. Choi D.-H. Kim J.-G. Lee I.-S. (2021). Endophytic fungi of salt-tolerant plants: diversity and ability to promote plant growth. J. Microbiol. Biotechnol. 31, 1526–1532. doi: 10.4014/jmb.2106.06007 34528914 PMC9705876

[B80] Khan A. L. Hamayun M. Ahmad N. Hussain J. Kang S. M. Kim Y. H. . (2011). Salinity stress resistance offered by endophytic fungal interaction between *Penicillium minioluteum* LHL09 and glycine max. l. Wold J. Microbiol. Biotechnol. 21 (9), 893–902. doi: 10.4014/JMB.1103.03012 21952365

[B81] Khan A. R. Ullah I. Waqas M. Shahzad R. Hong S.-J. Park G.-S. . (2015). Plant growth-promoting potential of endophytic fungi isolated from *Solanum nigrum* leaves. World J. Microbiol. Biotechnol. 31, 1461–1466. doi: 10.1007/s11274-015-1888-0 26081602

[B82] Khokhar I. Haider M. S. Mukhtar I. Ali A. Mushtaq S. Ashfaq M. (2013). Effect of *Penicillium* species culture filtrate on seedling growth of wheat. Int. J. Agric. Res. 3 (1), 24–29.

[B83] Kim J. M. To T. K. Matsui A. Tanoi K. Kobayashi N. I. Matsuda F. . (2017). Acetate-mediated novel survival strategy against drought in plants. Nat. Plants. 3, 17097. doi: 10.1038/NPLANTS.2017.97 28650429

[B84] Kröber M. Wibberg D. Grosch R. Eikmeyer F. Verwaaijen B. Chowdhury S. P. . (2014). Effect of the strain *Bacillus amyloliquefaciens* FZB42 on the microbial community in the rhizosphere of lettuce under field conditions analyzed by whole metagenome sequencing. Front. Microbiol. 5. doi: 10.3389/FMICB.2014.00252 PMC4033844 24904564

[B85] Kwak M. J. Kong H. G. Choi K. Kwon S. K. Song J. Y. Lee J. . (2018). Rhizosphere microbiome structure alters to enable wilt resistance in tomato. Nat. Biotech. 36 (11), 1100–1116. doi: 10.1038/NBT.4232 30412196

[B86] Lee S. Behringer G. Hung R. Bennett J. (2019a). Effects of fungal volatile organic compounds on *Arabidopsis thaliana* growth and gene expression. Fungal Ecol. 37, 1–9. doi: 10.1016/j.funeco.2018.08.004

[B87] Lee S. A. Kanth B. K. Kim H. S. Kim T. W. Sang M. K. Song J. . (2019b). Complete genome sequence of the plant growth-promoting endophytic bacterium *Rhodanobacter glycinis* T01E-68 isolated from tomato (*Solanum lycopersicum* l.) plant roots. Korean J. Microbiol. 55 (4), 422–424. doi: 10.7845/KJM.2019.9115

[B88] Lee K. E. Radhakrishnan R. Kang S. M. You Y. H. Joo G. J. Lee I. J. . (2015). *Enterococcus faecium* LKE12 cell-free extract accelerates host plant growth *via* gibberellin and indole-3-acetic acid secretion. World J. Microbiol. Biotechnol. 25 (9), 1467–1475. doi: 10.4014/JMB.1502.02011 25907061

[B89] Liu H. Brettell L. E. Qiu Z. Singh B. K. (2020). Microbiome-mediated stress resistance in plants. Trends Plant Sci. 25 (8), 733–743. doi: 10.1016/j.tplants.2020.03.014 32345569

[B90] Liu H. Qiu Z. Ye J. Verma J. P. Li J. Singh B. K. (2022). Effective colonisation by a bacterial synthetic community promotes plant growth and alters soil microbial community. J. Sustain. Agric. 1 (1), 30–42. doi: 10.1002/sae2.12008

[B91] Li J. Wang J. Liu H. Macdonald C. A. Singh B. K. (2022). Application of microbial inoculants significantly enhances crop productivity: A meta-analysis of studies from 2010 to 2020. J. Sust. Agric. Environ. 1 (3), 216–225. doi: 10.1002/sae2.12028

[B92] Macias-Benitez S. Garcia-Martinez A. M. Caballero Jimenez P. Gonzalez J. M. Tejada Moral M. Parrado Rubio J. (2020). Rhizospheric organic acids as biostimulants: Monitoring feedbacks on soil microorganisms and biochemical properties. Front. Plant Sci. 11. doi: 10.3389/FPLS.2020.00633 PMC7270406 32547578

[B93] Marin O. González B. Poupin M. J. (2021). From microbial dynamics to functionality in the rhizosphere: a systematic review of the opportunities with synthetic microbial communities. Front. Plant Sci. 12. doi: 10.3389/fpls.2021.650609 PMC8210828 34149752

[B94] Meena K. K. Kumar M. Kalyuzhnaya M. G. Yandigeri M. S. Singh D. P. Saxena A. K. . (2012). Epiphytic pink-pigmented methylotrophic bacteria enhance germination and seedling growth of wheat (*Triticum aestivum*) by producing phytohormone. Antonie van Leeuwenhoek. 101 (4), 777–786. doi: 10.1007/S10482-011-9692-9 22200783

[B95] Melo I. S. D. Piccinin E. (1999). Toxic metabolites from culture filtrate of *Fusarium oxysporum* and its effects on cucumber cells and plantlets. Rev. microbiologia. 30, 104–106. doi: 10.1590/S0001-37141999000200003

[B96] Middleton E. L. Rihardson S. Koziol L. Palmer C. E. Yermakov Z. Y. Henning J. A. . (2015). Locally adapted arbuscular mycorrhizal fungi improve vigor and resistance to herbivory of native prairie plant species. Ecosphere 61–, 16. doi: 10.1890/ES15-00152.1

[B97] Miransari M. (2011). Soil microbes and plant fertilization. Appl. Microbiol. Biotechnol. 92 (5), 875–885. doi: 10.1007/S00253-011-3521-Y 21989562

[B98] Morcillo R. J. L. Manzanera M. (2021). The effects of plant-associated bacterial exopolysaccharides on plant abiotic stress tolerance. Metabolites. 11 (6), 337. doi: 10.3390/METABO11060337 34074032 PMC8225083

[B99] Morcillo R. J. Singh S. K. He D. An G. Vílchez J. I. Tang K. . (2020). Rhizobacterium-derived diacetyl modulates plant immunity in a phosphate-dependent manner. EMBO J. 39 (2), e102602. doi: 10.15252/EMBJ.2019102602 31802519 PMC6960444

[B100] Murali M. Amruthesh K. N. (2015). Plant growth-promoting fungus *Penicillium oxalicum* enhances plant growth and induces resistance in pearl millet against downy mildew disease. J. Phytopathol. 163, 743–754. doi: 10.1111/jph.12371

[B101] Naamala J. Smith D. L. (2021). Microbial derived compounds, a step toward enhancing microbial inoculants technology for sustainable agriculture. Front. Microbiol. 12. doi: 10.3389/fmicb.2021.634807 PMC7930237 33679668

[B102] Noceto P. A. Bettenfeld P. Boussageon R. Hériché M. Sportes A. van Tuinen D. . (2021). Arbuscular mycorrhizal fungi, a key symbiosis in the development of quality traits in crop production, alone or combined with plant growth-promoting bacteria. Mycorrhiza. 31 (6), 655–669. doi: 10.1007/S00572-021-01054-1 34633544

[B103] Ogórek R. (2016). Enzymatic activity of potential fungal plant pathogens and the effect of their culture filtrates on seed germination and seedling growth of garden cress (*Lepidium sativum* l.). Eur. J. Plant Pathol. 145 (2), 469–481. doi: 10.1007/s10658-016-0860-7

[B104] Onofre-Lemus J. Hernández-Lucas I. Girard L. Caballero-Mellado J. ACC (2009). (1-aminocyclopropane-1-carboxylate) deaminase activity, a widespread trait in burkholderia species and its growth-promoting effect on tomato plants. Appl. Environ. Microbiol. 75, 6581–6590. doi: 10.1128/AEM.01240-09 19700546 PMC2765135

[B105] Ortíz-Castro R. Contreras-Cornejo H. A. Macías-Rodríguez L. López-Bucio J. (2009). The role of microbial signals in plant growth and development. Plant Signal. Behav. 4 (8), 701–712. doi: 10.4161/PSB.4.8.9047 19820333 PMC2801380

[B106] Oskiera M. Szczech M. Stępowska A. Smolińska U. Bartoszewski G. (2017). Monitoring of *Trichoderma* species in agricultural soil in response to application of biopreparations. Biol. Control. 113, 65–72. doi: 10.1016/j.biocontrol.2017.07.005

[B107] Papavizas G. C. (1982). Survival of *Trichoderma harzianum* in soil and in pea and bean rhizospheres. Phytopathology. 72 (1), 121. doi: 10.1094/PHYTO-72-121

[B108] Parnell J. J. Berka R. Young H. A. Sturino J. M. Kang Y. Barnhart D. M. . (2016). From the lab to the farm: An industrial perspective of plant beneficial microorganisms. Front. Plant Sci. 7. doi: 10.3389/FPLS.2016.01110 PMC4973397 27540383

[B109] Parveen S. Wani A. H. Bhat M. Y. (2019). Effect of culture filtrates of pathogenic and antagonistic fungi on seed germination of some economically important vegetables. Braz. J. Biol. Sci. 6 (12), 133–139. doi: 10.21472/bjbs.061212

[B110] Pellegrini M. Pagnani G. Bernardi M. Mattedi A. Spera D. M. del Gallo M. (2020). Cell-free supernatants of plant growth-promoting bacteria: a review of their use as biostimulant and microbial biocontrol agents in sustainable agriculture. Sustainability. 12 (23), 9917. doi: 10.3390/SU12239917

[B111] Pérez-Sánchez A. Singh S. Pérez-Sánchez E. J. Segura-Silva R. M. (2018). Techno-economic evaluation and conceptual design of a liquid biofertilizer plant. Rev. Colombiana Biotecnología 20 (2), 6–18. doi: 10.15446/rev.colomb.biote.v20n2.77053

[B112] Perin L. Martínez-Aguilar L. Paredes-Valdez G. Baldani J. I. Estrada-de los Santos P. Reis V. M. . (2006). *Burkholderia silvatlantica* sp. nov., a diazotrophic bacterium associated with sugar cane and maize. Int. J. Syst. Evol. Microbiol. 56 (8), 1931–1937. doi: 10.1099/IJS.0.64362-0 16902033

[B113] Pineda A. Dicke M. Pieterse C. M. J. Pozo M. J. (2013). Beneficial microbes in a changing environment: are they always helping plants to deal with insects? Funct. Ecol. 27 (3), 574–586. doi: 10.1111/1365-2435.12050

[B114] Posada L. F. Ramírez M. Ochoa-Gómez N. Cuellar-Gaviria T. Z. Argel-Roldan L. E. Ramírez C. A. . (2016). Bioprospecting of aerobic endospore-forming bacteria with biotechnological potential for growth promotion of banana plants. Sci. Hortic. 212, 81–90. doi: 10.1016/j.scienta.2016.09.040

[B115] Puri A. Padda K. P. Chanway C. P. (2020). Sustaining the growth of *Pinaceae* trees under nutrient-limited edaphic conditions *via* plant-beneficial bacteria. PloS One 2615 (8), e0238055. doi: 10.1371/journal.pone.0238055 PMC7449467 32845898

[B116] Rabhi N. E. H. Silini A. Cherif-Silini H. Yahiaoui B. Lekired A. Robineau M. . (2018). *Pseudomonas knackmussii* MLR6, a rhizospheric strain isolated from halophyte, enhances salt tolerance in arabidopsis thaliana. J. Appl. Microbiol. 125 (6), 1836–1851. doi: 10.1111/JAM.14082 30142236

[B117] Radhakrishnan R. Kang S. M. Baek I. Y. Lee I. J. (2014). Characterization of plant growth-promoting traits of *Penicillium* species against the effects of high soil salinity and root disease. J. Plant Interact. 9 (1), 754–762. doi: 10.1080/17429145.2014.930524

[B118] Radhakrishnan R. Shim K. B. Lee B. W. Hwang C. D. Pae S. B. Park C. H. . (2013). IAA-producing penicillium sp. NICS01 triggers plant growth and suppresses *Fusarium* sp.-induced oxidative stress in sesame (*Sesamum indicum* l.). World J. Microbiol. Biotechnol. 23 (6), 856–863. doi: 10.4014/JMB.1209.09045 23676921

[B119] Rahman M. M. Mostofa M. G. Rahman M. A. Islam M. R. Keya S. S. Das A. K. . (2019). Acetic acid: a cost-effective agent for mitigation of seawater-induced salt toxicity in mung bean. Sci. Rep. 9, 1–15. doi: 10.1038/s41598-019-51178-w 31645575 PMC6811677

[B120] Rahman M. A. Sultana R. Begum M. F. Alam M. F. (2012). Effect of culture filtrates of *Trichoderma* on seed germination and seedling growth in chili international journal of biosciences. Int. J. Biosci. 2 (4), 46–55.

[B121] Raithak P. V. Gachande B. D. (2013). Effect of culture filtrates of tomato plant pathogenic fungi on seed germination and seedling growth of tomato (*Lycopersicon esclentum* mill.). Curr. Botany. 4 (1), 9–11.

[B122] Rashnoo A. Movahedi Z. Rostami M. Ghabooli M. (2020). *Piriformospora indica* culture filtrate and biofertilizer (nitrokara) promote chicory (*Cichorium intybus* l.) growth and morpho-physiological traits in an aeroponic system and soil culture. Int. J. Hortic. Sci. Technol. 7, 353–363. doi: 10.22059/ijhst.2020.292414.324

[B123] Ravensberg W. J. (2011). A roadmap to the successful development and commercialization of microbial pest control products for control of arthropods. (Vol. 10). Springer Sci. Business Media. doi: 10.1007/978-94-007-0437-4

[B124] Reddy C. A. Saravanan R. S. (2013). Polymicrobial multi-functional approach for enhancement of crop productivity. Adv. Appl. Microbiol. 82, 53–113. doi: 10.1016/B978-0-12-407679-2.00003-X 23415153

[B125] Rodríguez H. Fraga R. Gonzalez T. Bashan Y. (2007). Genetics of phosphate solubilization and its potential applications for improving plant growth-promoting bacteria. First Int. Meeting Microbial Phosphate Solubilization 102, 15–21. doi: 10.1007/978-1-4020-5765-6_2

[B126] Rodríguez-Morgado B. Jiménez P. C. Moral M. T. Rubio J. P. (2017). Effect of l-lactic acid from whey wastes on enzyme activities and bacterial diversity of soil. Biol. Fertil. Soils. 53 (4), 389–396. doi: 10.1007/S00374-017-1187-Z

[B127] Rondina A. B. L. dos Santos Sanzovo A. W. Guimarães G. S. Wendling J. R. Nogueira M. A. Hungria M. (2020). Changes in root morphological traits in soybean co-inoculated with *Bradyrhizobium* spp. and *Azospirillum brasilense* or treated with *A. brasilense* exudates. Biol. Fertil. Soils. 56 (4), 537–549. doi: 10.1007/s00374-020-01453-0

[B128] Ryu C. M. Faragt M. A. Hu C. H. Reddy M. S. Wei H. X. Paré P. W. . (2003). Bacterial volatiles promote growth in arabidopsis. Proc. Natl. Acad. Sci. U.S.A. 100 (8), 4927–4932. doi: 10.1073/PNAS.0730845100 12684534 PMC153657

[B129] Saber W. I. A. Ghoneem K. M. Rashad Y. M. Al-Askar A. A. (2017). *Trichoderma harzianum* WKY1: an indole acetic acid producer for growth improvement and anthracnose disease control in sorghum. Biocontrol Sci. Technol. 27 (5), 654–676. doi: 10.1080/09583157.2017.1321733

[B130] Saha M. Sarkar S. Sarkar B. Sharma B. K. Bhattacharjee S. Tribedi P. (2016). Microbial siderophores and their potential applications: a review. Environ. Sci. pollut. Res. 23 (5), 3984–3999. doi: 10.1007/S11356-015-4294-0 25758420

[B131] Sánchez-López Á.M. Baslam M. de Diego N. Muñoz F. J. Bahaji A. Almagro G. . (2016). Volatile compounds emitted by diverse phytopathogenic microorganisms promote plant growth and flowering through cytokinin action. Plant Cell Environ. 39 (12), 2592–2608. doi: 10.1111/PCE.12759 27092473

[B132] Sasse J. Martinoia E. Northen T. (2018). Feed your friends: do plant exudates shape the root microbiome? Trends Plant Sci. 23 (1), 25–41. doi: 10.1016/J.TPLANTS.2017.09.003 29050989

[B133] Schmidt R. Köberl M. Mostafa A. Ramadan E. M. Monschein M. Jensen K. B. . (2014). Effects of bacterial inoculants on the indigenous microbiome and secondary metabolites of chamomile plants. Front. Microbiol. 5. doi: 10.3389/fmicb.2014.00064 PMC3928675 24600444

[B134] Schulz-Bohm K. Martín-Sánchez L. Garbeva P. (2017). Microbial volatiles: small molecules with an important role in intra- and interkingdom interactions. Front. Microbiol. 8. doi: 10.3389/fmicb.2017.02484 PMC5733050 29312193

[B135] Shayanthan A. Ordoñez P. A. C. Oresnik I. J. (2022). The role of synthetic microbial communities (SynCom) in sustainable agriculture. Front. Agron. 4. doi: 10.3389/fagro.2022.896307

[B136] Sirrenberg A. Göbel C. Grond S. Czempinski N. Ratzinger A. Karlovsky P. . (2007). *Piriformospora indica* affects plant growth by auxin production. Physiol. Plant 131 (4), 581–589. doi: 10.1111/J.1399-3054.2007.00983.X 18251849

[B137] Sitaraman R. (2015). *Pseudomonas* spp. as models for plant-microbe interactions. Front. Plant Sci. 6. doi: 10.3389/fpls.2015.00787 PMC4586426 26483805

[B138] Spaepen S. Vanderleyden J. Remans R. (2007). Indole-3-acetic acid in microbial and microorganism-plant signaling. FEMS Microbiol. Rev. 31 (4), 425–448. doi: 10.1111/J.1574-6976.2007.00072.X 17509086

[B139] Spinelli V. Brasili E. Sciubba F. Ceci A. Giampaoli O. Miccheli A. . (2022). Biostimulant effects of *Chaetomium globosum* and *Minimedusa polyspora* culture filtrates on *Cichorium intybus* plant: growth performance and metabolomic traits. Front. Plant Sci. 13. doi: 10.3389/fpls.2022.879076 PMC9134003 35646045

[B140] Sung G. H. Shrestha B. Park K. B. Han S. K. Sung J. M. (2011). Enhancing effect of *Shimizuomyces paradoxus* on seed germination and seedling growth of canola, plant growth of cucumber, and harvest of tomato. Mycobiology. 39 (1), 7. doi: 10.4489/MYCO.2011.39.1.007 22783066 PMC3385086

[B141] Svenningsen N. B. Watts-Williams S. J. Joner E. J. Battini F. Efthymiou A. Cruz-Paredes C. . (2018). Suppression of the activity of arbuscular mycorrhizal fungi by the soil microbiota. ISME J. 12 (5), 1296–1307. doi: 10.1038/s41396-018-0059-3 29382946 PMC5931975

[B142] Tallapragada P. Dikshit R. Seshagiri S. (2015). Isolation and optimization of IAA producing *Burkholderia seminalis* and its effect on seedlings of tomato. Songklanakarin J. Sci. Technol. 37 (5), 553–559.

[B143] Trujillo-Roldán M. A. Valdez-Cruz N. A. González-Monterrubio C. F. Acevedo-Sánchez E. V. Martínez-Salinas C. García-Cabrera R. I. . (2013). Scale-up from shake flasks to pilot-scale production of the plant growth-promoting bacterium *Azospirillum brasilense* for preparing a liquid inoculant formulation. Appl. Microbiol. Biotechnol. 97, 9665–9674. doi: 10.1007/s00253-013-5199-9 24061414

[B144] Tsavkelova E. A. Klimova S. Y. Cherdyntseva T. A. Netrusov A. I. (2006a). Microbial producers of plant growth stimulators and their practical use: A review. Appl. Biochem. Microbiol. 42 (2), 117–126. doi: 10.1134/S0003683806020013 16761564

[B145] Tsavkelova E. A. Klimova S. Y. Cherdyntseva T. A. Netrusov A. I. (2006b). Hormones and hormone-like substances of microorganisms: a review. Appl. Biochem. Microbiol. 42 (3), 229–235. doi: 10.1134/S000368380603001X 16878539

[B146] Utsumi Y. Utsumi C. Tanaka M. Ha C. Takahashi S. Matsui A. . (2019). Acetic acid treatment enhances drought avoidance in cassava (*Manihot esculenta* crantz). Front. Plant Sci. 10. doi: 10.3389/FPLS.2019.00521 PMC6492040 31105723

[B147] Vankudoth K. R. Sivadeveuni G. Reddy S. M. (2015). Influence of different species of *Penicillium* and their culture filtrates on seed germination and seedling growth of sorghum. J. Biochem. Technol. 5 (4), 832–837.

[B148] Varma A. Verma S. Sudha, Sahay N. Bütehorn B. Franken P. (1999). *Piriformospora indica*, a cultivable plant-growth-promoting root endophyte. Appl. Environ. Microbiol. 65 (6), 2741–2744. doi: 10.1128/AEM.65.6.2741-2744.1999 10347070 PMC91405

[B149] Vassilev N. Vassileva M. Martos V. Garcia del Moral L. F. Kowalska J. Tylkowski B. . (2020). Formulation of microbial inoculants by encapsulation in natural polysaccharides: focus on beneficial properties of carrier additives and derivatives. Front. Plant Sci. 11. doi: 10.3389/FPLS.2020.00270 PMC7077505 32211014

[B150] Vílchez J. I. Yang Y. He D. Zi H. Peng L. Lv S. . (2020). DNA Demethylases are required for myo-inositol-mediated mutualism between plants and beneficial rhizobacteria. Nat. Plants. 6 (8), 983–995. doi: 10.1038/S41477-020-0707-2 32661278

[B151] Wang Y. Zhao A. Morcillo R. J. L. Yu G. Xue H. Rufian J. S. . (2021). A bacterial effector protein uncovers a plant metabolic pathway involved in tolerance to bacterial wilt disease. Mol. Plant 14 (8), 1281–1296. doi: 10.1016/J.MOLP.2021.04.014 33940211

[B152] Waqas M. Kim Y. H. Khan A. L. Shahzad R. Asaf S. Hamayun M. . (2017). Additive effects due to biochar and endophyte application enable soybean to enhance nutrient uptake and modulate nutritional parameters. J. Zhejiang Univ. Sci. 18 (2), 109. doi: 10.1631/JZUS.B1500262 PMC5296228 28124840

[B153] Wu Y. He Y. Yin H. Chen W. Wang Z. Xu L. . (2012). Isolation of phosphate-solubilizing fungus and its application in solubilization of rock phosphates. Pakistan J. Biol. Sci. PJBS 15, 1144–1151. doi: 10.3923/pjbs.2012.1144.1151 24261118

[B154] Wu L. Li X. Ma L. Borriss R. Wu Z. Gao X. (2018). Acetoin and 2,3-butanediol from *Bacillus amyloliquefaciens* induce stomatal closure in *Arabidopsis thaliana* and *Nicotiana benthamiana* . J. Exp. Bot. 69 (22), 5625–5635. doi: 10.1093/jxb/ery326 30295868

[B155] Yakhin O. I. Lubyanov A. A. Yakhin I. A. Brown P. H. (2017). Biostimulants in plant science: A global perspective. Front. Plant Sci. 7. doi: 10.3389/FPLS.2016.02049 PMC5266735 28184225

[B156] Yandigeri M. S. Meena K. K. Singh D. Malviya N. Singh D. P. Solanki M. K. . (2012). Drought-tolerant endophytic actinobacteria promote growth of wheat (*Triticum aestivum*) under water stress conditions. Plant Growth Regul. 68 (3), 411–420. doi: 10.1007/S10725-012-9730-2

[B157] You Y.-H. (2012). Fungal diversity and plant growth promotion of endophytic fungi from six halophytes in suncheon bay. J. Microbiol. Biotechnol. 22, 1549–1556. doi: 10.4014/jmb.1205.05010 23124347

[B158] Yuan J. Zhao M. Li R. Huang Q. Raza W. Rensing C. . (2017). Microbial volatile compounds alter the soil microbial community. Environ. Sci. pollut. Res. 24 (28), 22485–22493. doi: 10.1007/s11356-017-9839-y 28803260

[B159] Zhalnina K. Louie K. B. Hao Z. Mansoori N. da Rocha U. N. Shi S. . (2018). Dynamic root exudate chemistry and microbial substrate preferences drive patterns in rhizosphere microbial community assembly. Nat. Microbiol. 3 (4), 470–480. doi: 10.1038/S41564-018-0129-3 29556109

[B160] Zhang H. Kim M. S. Krishnamachari V. Payton P. Sun Y. Grimson M. . (2007). Rhizobacterial volatile emissions regulate auxin homeostasis and cell expansion in arabidopsis. Planta. 226 (4), 839–851. doi: 10.1007/S00425-007-0530-2 17497164

[B161] Zhang R. Ouyang J. Xu X. Li J. Rehman M. Deng G. . (2022). Nematicidal activity of *Burkholderia arboris* J211 against *Meloidogyne incognita* on tobacco. Front. Microbiol 13, 915546. doi: 10.3389/fmicb.2022.915546 35756018 PMC9226767

[B162] Zhang H. Sun Y. Xie X. Kim M. S. Dowd S. E. Paré P. W. (2009). A soil bacterium regulates plant acquisition of iron *via* deficiency-inducible mechanisms. Plant J. 58 (4), 568–577. doi: 10.1111/J.1365-313X.2009.03803.X 19154225

[B163] Zhang H. Xie X. Kim M. S. Kornyeyev D. A. Holaday S. Paré P. W. (2008). Soil bacteria augment arabidopsis photosynthesis by decreasing glucose sensing and abscisic acid levels in planta. Plant J. 56 (2), 264–273. doi: 10.1111/J.1365-313X.2008.03593.X 18573192

[B164] Zhong Y. Yang Y. Liu P. Xu R. Rensing C. Fu X. . (2019). Genotype and rhizobium inoculation modulate the assembly of soybean rhizobacterial communities. Plant Cell Environ. 42 (6), 2028–2044. doi: 10.1111/pce.13519 30646427

[B165] Zhou J.-Y. Li X. Zheng J.-Y. Dai C.-C. (2016). Volatiles released by endophytic *Pseudomonas fluorescens* promoting the growth and volatile oil accumulation in. Atractylodes lancea. Plant Physiol. Biochem. 101, 132–140. doi: 10.1016/j.plaphy.2016.01.026 26874622

